# The B169L protein of African swine fever virus functions as a viroporin that activates the calcium-mediated inflammasome

**DOI:** 10.1371/journal.ppat.1013686

**Published:** 2025-11-14

**Authors:** Jiaqi Li, Qiguang Li, Yanjin Wang, Zhanhao Guo, Yuxin Qu, Xiao Wang, Hao Deng, Jingwen Dai, Lian-Feng Li, Wen-Rui He, Haojie Ren, Zhaobing Gao, Bingqing Xia, Su Li, Hua-Ji Qiu

**Affiliations:** 1 State Key Laboratory of Animal Disease Prevention and Control, National African Swine Fever Para-Reference Laboratory, National High-Containment Facilities for Animal Diseases Control and Prevention, Harbin Veterinary Research Institute, Chinese Academy of Agricultural Sciences, Harbin, Heilongjiang, China; 2 CAS Key Laboratory of Receptor Research, State Key Laboratory of Drug Research, Shanghai Institute of Materia Medica, Chinese Academy of Sciences, Shanghai, China; 3 School of Basic Medical Sciences, Binzhou Medical University, Yantai, Shandong, China; 4 International Joint Research Center for National Animal Immunology, College of Veterinary Medicine, Henan Agricultural University, Zhengzhou, Henan, China; Johns Hopkins University School of Medicine, UNITED STATES OF AMERICA

## Abstract

African swine fever (ASF) is a highly contagious and often fatal viral disease caused by African swine fever virus (ASFV), which poses a significant economic burden on the global pig industry. ASFV infection triggers a robust production of proinflammatory cytokines, leading to severe inflammation that contributes significantly to the high mortality rate associated with ASF. However, the underlying mechanisms remain incompletely understood. Here, we identified the ASFV B169L protein (pB169L) as a viroporin that exerts dual functions in viral replication and proinflammatory responses. We demonstrated that pB169L formed oligomeric calcium (Ca^2+^)-permeable channels *in vitro* by bilayer lipid membrane assay. The ectopically expressed pB169L significantly altered Ca^2+^ homeostasis in cells and induced robust proinflammatory responses. Mutagenesis revealed critical residues—including P29, K55, and K57—that are indispensable for channel function and proinflammatory signaling. Importantly, the *B169L* gene knockdown during ASFV infection reduced inflammasome activation and viral replication, highlighting its dual role as both a structural component of virus and an inflammatory mediator. These findings provide the first direct evidence that ASFV encodes a functional viroporin and uncover a novel mechanism by which ASFV manipulates Ca^2+^ homeostasis to drive inflammasome activation, offering new insights into ASFV pathogenesis and potential antiviral targets.

## Author summary

African swine fever (ASF) is a devastating infectious disease of domestic pigs and wild boar caused by African swine fever virus (ASFV), leading to great socioeconomic losses to the pig industry worldwide. The robust inflammatory responses elicited by ASFV infection is a critical contributor to mortality in pigs. However, the mechanism underlying the proinflammatory responses induced by ASFV infection is not yet fully understood. In this study, we demonstrated that the ASFV B169L protein (pB169L) is a Ca^2+^-permeable cation channel protein that disrupts Ca^2+^ homeostasis. This disruption activates the NLRP3 inflammasome, a key trigger of proinflammatory cytokine secretion. Importantly, P29A, K55A, and K57A mutations abolished the pB169L-mediated NLRP3 inflammasome activation by inhibiting pB169L oligomerization and reducing calcium flux, respectively. Furthermore, *B169L* knockdown inhibits the production of the proinflammatory cytokine interleukin1beta (IL-1*β*) and IL-18 during ASFV infection. Our findings reveal a previously unrecognized role of *B169L* in ASFV pathogenesis and highlight the viroporin as potential targets for therapeutic intervention against ASF.

## Introduction

African swine fever (ASF) is a highly contagious and often fatal viral disease of pigs. As an animal disease notifiable to the World Organization for Animal Health (WOAH), ASF was initially endemic in Kenya and subsequently spread across Africa, Europe, and Asia, threatening the global pig industry [1]. African swine fever virus (ASFV), the causative agent of ASF, belongs to the genus *Asfivirus* of the family *Asfarviridae*. ASFV is a large, enveloped, double-stranded DNA (dsDNA) virus, with virions ranging in diameter from 260 to 300 nm [2]. The virion exhibits a complex multilayered structure, consisting of an outer envelope, a capsid, an inner envelope, a core shell, and a nucleoid [2]. The ASFV genome, approximately 170−194 kilobase pairs (kb) in size, encodes 68 structural proteins and over 100 non-structural proteins [2]. The structural proteins of ASFV are not only essential for virion morphogenesis but also involved in the modulation of innate immunity. Among the multiple host responses to ASFV infection, proinflammatory responses play key roles in pathogenesis. However, the limited understanding of the functions of these proteins has significantly hindered the development of vaccines and antivirals.

Several proinflammatory cytokines, including interleukin 1beta (IL-1*β*), IL-18, and tumor necrosis factor alpha (TNF-*α)*, are critical for antiviral immunity. ASFV infection induces robust cytokine storms in pigs, leading to the subsequent hemorrhagic lesions of tissues and the depletion of immune cells, which is one of the leading causes of death [3]. Recently, we have demonstrated that ASFV infection induces the expression of proinflammatory cytokines in primary porcine alveolar macrophages (PAMs) [4]. The inflammasome serves as a crucial signaling pathway in the proinflammatory responses. The inflammasomes can be activated by multiple stimuli, including viral RNAs [5] and disruption in ion homeostasis [6].

Accumulating evidence shows that viroporins, which can affect membrane permeability and disrupt ion homeostasis in cellular compartments, serve as crucial activation signals for the NLRP3 inflammasome, resulting in secretion of IL-1*β* and IL-18 [7]. Viroporins are small, virus-encoded proteins with high hydrophobicity, which usually oligomerize in cellular membranes and disrupt ion homeostasis [8]. They are also involved in multiple stages of the viral life cycle, with the primary role involved in the assembly and release of virions from infected cells [9,10]. The hepatitis C virus (HCV) p7 forms cation channels in artificial membranes and is involved in viral assembly [11,12]. Viroporins also regulate various cellular processes, including modulating membrane permeability, ion homeostasis, membrane remodeling, and glycoprotein trafficking [8]. Of all biologically relevant ions, Ca^2+^ serves as a second messenger and directly modulates many cellular processes, including NLRP3 inflammasome activation [6]. Viroporins can increase intracellular Ca^2+^ concentration by facilitating the influx of extracellular calcium or calcium leakage from intracellular calcium reservoirs [13]. Several viral proteins, including the SARS-CoV-2 E protein, influenza A virus (IAV) M2 protein, and encephalomyocarditis virus (EMCV) 2B protein, have been identified as key mediators of the NLRP3 inflammasome activation [13–17].

The ASFV B169L protein (pB169L) is a membrane-associated protein. A recent study has shown that the hairpin transmembrane domain of pB169L exhibits viroporin-like activity [18]. However, the potential of full-length pB169L to function as a viroporin remains unclear. Furthermore, the involvement of pB169L in viral pathogenesis has not yet been elucidated. In this study, we demonstrate that the proinflammatory responses induced by pB169L depend on its viroporin activity, with residues P29, K55, and K57 identified as critical determinants. Targeting the pB169L channel represents a promising antiviral strategy and provides insights for developing ASF vaccines.

## Results

### The ASFV pB169L is able to activate the inflammasome pathway

**Fig 1:**
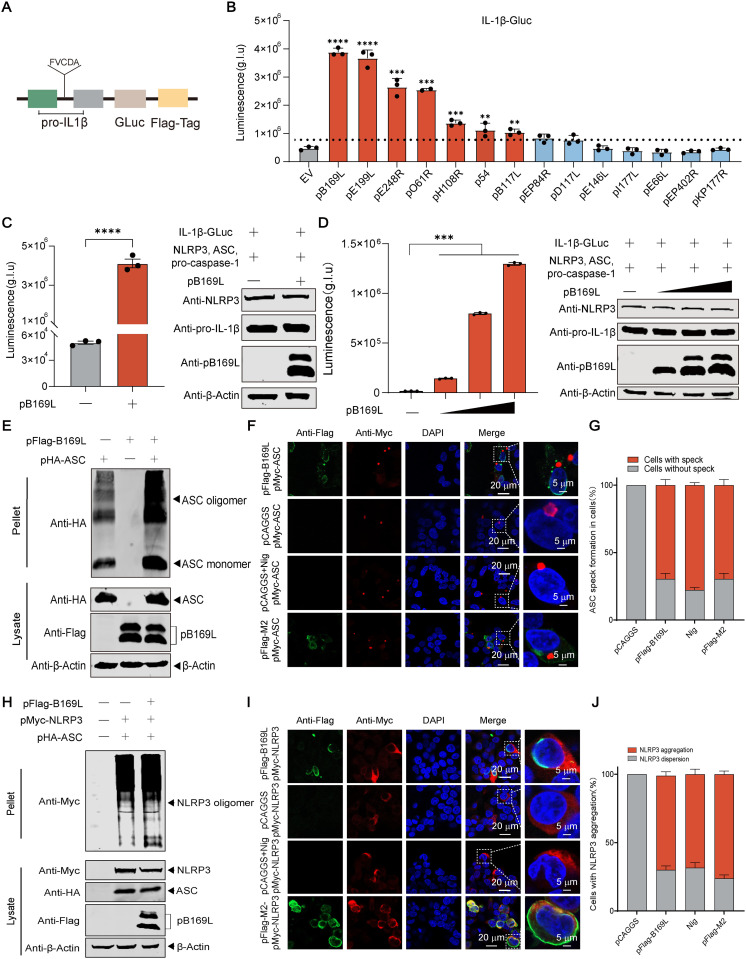
The B169L protein (pB169L) of ASFV activates the NLRP3 inflammasome. **(A)** Schematic of pro-interleukin 1beta (pro-IL-1*β*)-*Gaussia* luciferase (iGLuc) reporter. **(B)** Screening for ASFV structural proteins that activate the NLRP3 inflammasome. HEK293T cells were cotransfected with the iGLuc-based NLRP3 inflammasome system (300 ng of pFlag-pro-IL-1*β*-Gluc (pFlag-iGluc), 10 ng of pFlag-pro-caspase-1, 10 ng of pFlag-ASC, and 60 ng of pFlag-NLRP3) and the plasmids expressing the structural proteins of ASFV or the empty vector pCAGGS, and the luciferase activity in the supernatants were examined at 24 hours posttransfection (hpt). (**C** and **D**) pB169L activates the inflammasome in a dose-dependent manner. HEK293T cells were transfected with increasing doses of pFlag-B169L or pCAGGS as well as the NLRP3-dependent inflammasome reporter as described above, and the luciferase activity of the supernatants and the intracellular protein expression levels were evaluated at 24 hpt. **(E)** pB169L promotes ASC oligomerization. HEK293T cells were cotransfected with pHA-ASC and pFlag-B169L or pCAGGS. At 24 hpt, the cells were incubated with 200 mM dioctanoyl di-succinimidyl ester (DSS) (final concentration: 4 μM) at 4°C for 30 minutes. The cells were incubated with 50 µL of 2 × protein loading buffer. ASC oligomerization was evaluated by Western blotting analysis. (**F** and **G**) pB169L promotes the speck formation of ASC. HEK293T cells were cotransfected with pMyc-ASC and either pFlag-B169L, pFlag-M2, or pCAGGS. At 24 hpt, the cells were incubated with anti-Flag and anti-Myc antibodies, followed by incubation with the 488- or 633-conjugated secondary antibodies for 50 minutes and the nuclei were stained with 4,6-diamidino-2-phenylindole (DAPI). Subsequently, fluorescence signals were examined by laser confocal microscopy (LCM). Scale bar, 20 µm. The percentage of ASC speck-positive cells was quantified by analyzing ten random microscopic fields. **(H)** pB169L promotes NLRP3 oligomerization. HEK293T cells were cotransfected with pMyc-NLRP3, pHA-ASC and either pFlag-B169L or pCAGGS. At 24 hpt, the cells were incubated with 200 mM DSS (final concentration: 4 μM) at 4°C for 30 minutes. The cells were incubated with 50 µL of 2 × protein loading buffer. NLRP3 oligomerization was evaluated by Western blotting analysis. **(I and J)** pB169L promotes the aggregation of NLRP3. HEK293T cells were cotransfected with pMyc-NLRP3 and either pFlag-B169L, pFlag-M2, or pCAGGS. At 24 hpt, the cells were fixed with 4% paraformaldehyde for 30 minutes, treated with 0.15% Triton X-100 for 15 minutes, incubated with anti-Flag and anti-Myc antibodies for 2 hours, and then incubated with the 488- or 633-conjugated secondary antibodies for 50 minutes. Finally, the fluorescence was observed using LCM. Scale bar, 20 μm. The percentage of NLRP3 aggregate-positive cells was quantified by randomly analyzing ten microscopic fields. All the data were analyzed using the Student’s *t* test. **, *P* < 0.01, ***, *P* < 0.001; ns, not significant, *P* ≥ 0.05.

To investigate the effects of the ASFV structural proteins on the proinflammatory responses, an NLRP3-dependent pro-IL-1*β*-Gaussia luciferase (iGLuc) reporter (Fig 1A) was used to screen for viral structural proteins involved in inflammasome activation. Among the tested viral proteins, pB169L was identified as a robust inducer of the NLRP3 inflammasome (Fig 1B). To further clarify the functional role of pB169L in the NLRP3-dependent inflammasome activation, we assessed the effects of pB169L on the activation of NLRP3-dependent iGLuc activity. The results demonstrated that pB169L significantly enhanced iGLuc activity in a dose-dependent manner (*P* < 0.0001) (Fig 1C and 1D).

Oligomerization and speck formation of apoptosis-associated speck-like protein containing a CARD (ASC) are pivotal molecular events in the inflammasome activation, which serve to amplify inflammatory signals via a cascade mechanism and coordinate the innate immune response. To further clarify the pathway through which pB169L activates the NLRP3 inflammasome, we assessed its effects on ASC oligomerization and speck formation. To investigate the effects of pB169L on ASC oligomerization, we performed a dioctanoyl di-succinimidyl ester (DSS) cross-linking assay. HEK293T cells were cotransfected with pFlag-B169L and pHA-ASC to assess ASC oligomer formation. The DSS cross-linking assay showed that the ectopically expressed pB169L promoted ASC oligomerization (Fig 1E). Additionally, confocal microscopy demonstrated that pB169L promoted ASC speck formation, as reflected by the quantification of speck-positive cells in ten randomly selected fields (Fig 1F and 1G). NLRP3 oligomerization is a critical step in inflammasome assembly. To further clarify the molecular mechanism underlying pB169L-induced NLRP3 inflammasome activation, HEK293T cells cotransfected with pMyc-NLRP3 and either pFlag-B169L or pCAGGS were analyzed for NLRP3 oligomerization using DSS cross-linking assay or laser confocal microscopy, respectively. As shown in Fig 1H, the ectopically expressed pB169L enhanced NLRP3 oligomerization, while laser confocal microscopy demonstrated that the pB169L expression recruited NLRP3 and promoted its aggregation (Fig 1I and 1J). However, no significant colocalization between pB169L and either NLRP3 or ASC was observed. These findings suggest that pB169L, as a key regulator of the NLRP3 inflammasome activation, may facilitate the assembly of its core components via indirect regulatory mechanisms or changes in the intracellular microenvironment.

### The ASFV pB169L mediates inflammasome activation by driving ion gradients

**Fig 2:**
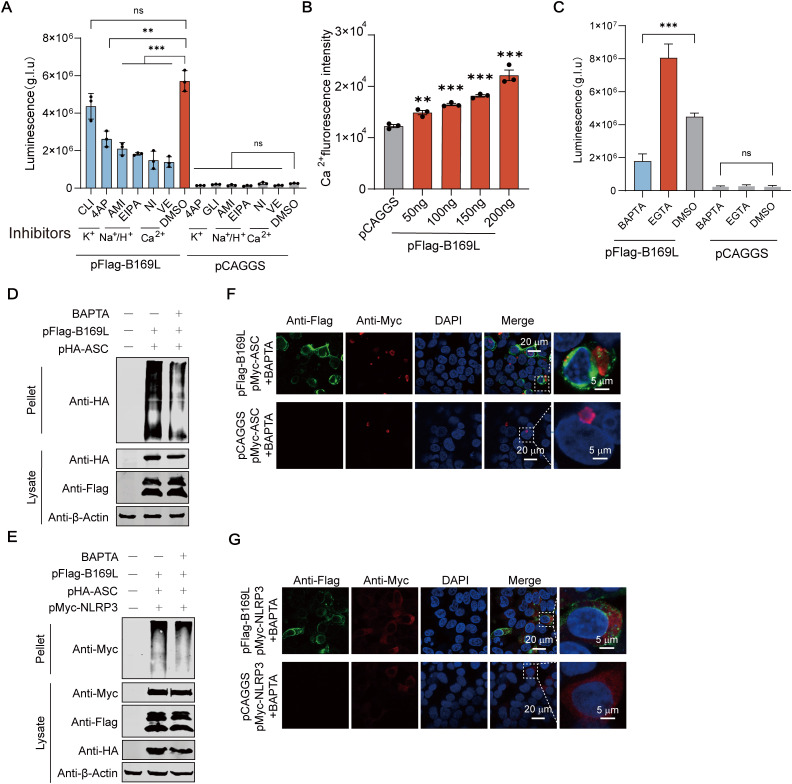
The B169L protein (pB169L) of ASFV activates inflammasome through driving ion levels. **(A)** Ca^2+^ inhibitors inhibit the activation of the NLRP3 inflammasome induced by pB169L. HEK293T cells were cotransfected with the iGLuc-based NLRP3 inflammasome system (300 ng of pFlag-pro-IL-1*β*-Gluc (pFlag-iGluc), 10 ng of pFlag-pro-caspase-1, 10 ng of pFlag-ASC, and 60 ng of pFlag-NLRP3) and the plasmids expressing the structural proteins of ASFV or the empty vector pCAGGS. At 12 hours posttransfection (hpt), the cells were treated with either EIPA, amiloride, verapamil, nifedipine, 4-AP, or glibenclamide for 12 hours. The supernatant luciferase activity was examined at 24 hpt. **(B)** pB169L dose-dependent promotes intracellular calcium ion increase. pFlag-B169L was transfected into HEK293T cells at different doses (50, 100, 150, or 200ng), and Flou-4 was added 24 hours later which were examined using a Fluo-4 Flux analysis kit. **(C)** The Ca^2+^-chelating agent BAPTA inhibits the activation of the NLRP3 inflammasome induced by pB169L. HEK293T cells were transfected with pFlag-B169L or pCAGGS as well as the NLRP3-dependent inflammasome reporter as described above. At 12 hpt, the cells were treated with BAPTA-AM (a quick-speed Ca^2+^ chelator) or EGTA for 12 hours. The supernatant luciferase activity was examined at 24 hpt. **(D)** BAPTA inhibits the pB169L-induced ASC oligomerization. HEK293T cells were cotransfected with pHA-ASC and either pFlag-B169L or pCAGGS. BAPTA-AM was added at 12 hpt. At 24 hpt, the cells were incubated with 200 mM dioctanoyl di-succinimidyl ester (DSS) (final concentration: 4 μM) at 4°C for 30 minutes. The cells were incubated with 50 µL of 2 × protein loading buffer. ASC oligomerization was evaluated by Western blotting analysis. **(E)** BAPTA inhibits the pB169L-induced NLRP3 oligomerization. HEK293T cells were cotransfected with pMyc-NLRP3, pHA-ASC, and either pFlag-B169L or pCAGGS. BAPTA-AM was added at 12 hpt. At 24 hpt, the cells were incubated with 200 mM DSS (final concentration: 4 μM) at 4°C for 30 minutes. The cells were incubated with 50 µL of 2 × protein loading buffer. NLRP3 oligomerization was evaluated by Western blotting analysis. **(F and G)** BAPTA inhibits the pB169L-induced ASC speck and the formation of NLRP3 aggregates. HEK293T cells were cotransfected with pFlag-B169L and either pMyc-ASC or pMyc-NLRP3. At 12 hpt, the cells were subsequently treated with BAPTA-AM for another 12 hours. The fluorescence was observed using laser confocal microscope. Scale bar, 20 µm. The data shown were from three independent experiments. The significance of the difference between the groups (*n* = 3) was determined using the Student’s *t* test (*, *P* < 0.05, **, *P* < 0.01, ***, *P* < 0.001; ns, not significant, *P* ≥ 0.05).

We have shown that pB169L activates the inflammasome and facilitates the release of IL-1*β*. Ion gradients—including those of sodium (Na^+^), potassium (K^+^), and calcium (Ca^2+^)—play essential roles in inflammasome activation; changes in these ion fluxes are critical for the initiation of inflammasome assembly and subsequent proinflammatory responses [19]. Therefore, we hypothesized that pB169L activates proinflammatory responses by disrupting intracellular ion homeostasis. At the cellular level, we examined the effects of ions on pB169L-induced NLRP3-dependent iGLuc reporter activation using six inhibitors targeting four ion-related pathways (Na^+^/H^+^, K^+^, and Ca^2+^), showing that the calcium channel inhibitors exhibited the robust inhibitory effects on the IL-1*β-*GLuc secretion compared with other channel inhibitors (Fig 2A), highlighting the critical role of calcium permeability in pB169L-mediated signaling. All inhibitors were used at safe concentrations (S1A−S1F Fig). Furthermore, we assessed the intracellular Ca^2+^ levels using Fluo-4, and the results revealed that the ectopically expressed pB169L enhanced intracellular Ca^2+^ level (Fig 2B). To investigate the role of Ca^2+^ in pB169L-induced NLRP3 inflammasome activation, we used the calcium chelators BAPTA-AM (a fast-acting Ca^2+^ chelator) and EGTA-AM (a slow-acting Ca^2+^ chelator) to inhibit calcium flux, and then analyzed pB169L-induced inflammasome activation. The results showed that BAPTA-AM, but not EGTA-AM, inhibited the activation of the inflammasome induced by pB169L (Fig 2C), indicating that rapid local changes in the Ca^2+^ concentration, rather than steady-state changes in the Ca^2+^ concentration, are involved in pB169L-induced inflammasome activation. All the inhibitors were used at safe concentrations (S1G and S1H Fig). Furthermore, these results revealed that BAPTA-AM treatment impaired the oligomerization of ASC and NLRP3 (Fig 2D and 2E), as well as ASC speck formation (Fig 2F) and NLRP3 aggregation (Fig 2G). Collectively, those data show that pB169L activates the inflammasome by disrupting Ca^2+^.

### The ASFV pB169L displays the characteristics of viroporins

**Fig 3:**
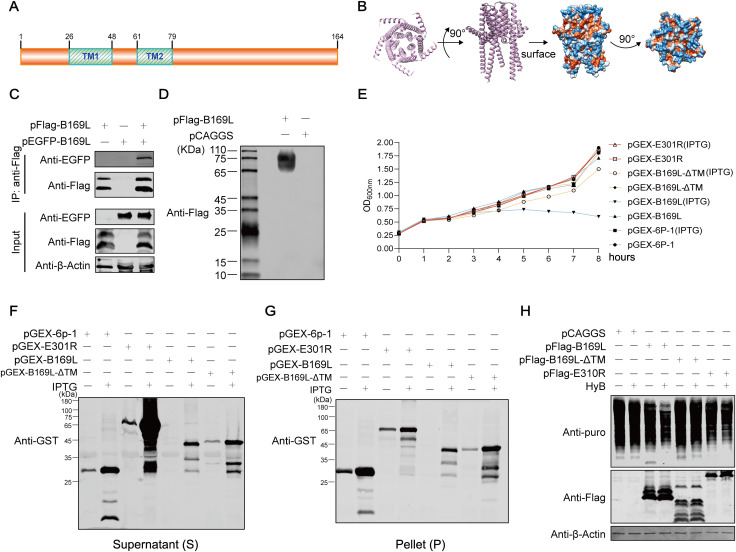
The B169L protein (pB169L) of ASFV exhibits the characteristics of viroporins. **(A)** A schematic diagram of the pB169L domains. The figure was generated by using the online software IBS 2.0 (https://www.ibs.renlab.org/). **(B)** Predicted multimeric structure of pB169L generated by AlphaFold2. **(C and D)** The oligomeric state of pB169L. HEK293T cells were cotransfected with pFlag-B169L and pEGFP-B169L (2 μg/well). At 24 hours posttransfection (hpt), the cells were lysed, and the resulting whole cell lysates (WCLs) were subjected to coimmunoprecipitation (co-IP) assay using the anti-Flag antibodies, followed by Western blotting analysis. as above (C). HEK293T cells were transfected with pFlag-B169L or pCAGGS. At 24 hpt, the cells were lysed, and the resulting WCLs were analyzed by native-PAGE as above (D). (**E–G**) pB169L inhibits the growth of *Escherichia coli*. The *E. coli* BL21(DE3) pLys cells transformed with pGEX-B169L, pGEX-B169L-ΔTM, or pGEX-E301R were grown to an OD value of 0.4 to 0.6 at 600 nm (OD_600nm_). Subsequently, the bacterial was divided into two equal portions and subjected to induction with 0.8 mM IPTG at 16°C or without IPTG induction under the same conditions. Cell growth was monitored by measuring OD_600nm_ (*n* ≥ 3). Finally, the supernatant (F) and pellet (G) fractions obtained after cell lysis were subjected to Western blotting analysis with anti-GST antibodies. **(H)** pB169L can enhance membrane permeability. Approximately 2 × 10^5^ HEK293T cells were transfected with pCAGGS, pFlag-B169L, pFlag-B169L-ΔTM, or pFlag-E301R. At 24 hpt, the cells were treated with 25 μg/mL HygB and 5 μg/mL puromycin. Protein synthesis and protein expression were analyzed by Western blotting with anti-puromycin and anti-Flag antibodies.

Viroporins are virus-encoded proteins that can assemble into oligomers and form ion channels in the organelle membrane, disrupting ion homeostasis and thereby triggering the inflammasome activation [7]. We hypothesized that pB169L may function as a viroporin. We then analyzed whether pB169L exhibits the characteristics of viroporins by combining structure modeling with biochemical assays. The ASFV pB169L consists of 164 amino acids and was predicted to contain two transmembrane (TM) domains (Figs 3A and S2A). Notably, multiple sequence alignment showed a high degree of conservation of the TM regions (aa 26–48 and 61–79) of pB169L across various ASFV strains (S2B Fig). Structural modeling revealed that pB169L assembled into a pentameric structure with high-confidence, adopting a funnel-shaped architecture (Fig 3B). The inner lining of the predicted channel was composed of a hydrophobic *α*-helix (sequence: PFIVALIITAVVLVVFFAIC), which surprisingly formed a hydrophilic pore within the hydrophobic tunnel (S3 Fig). To determine whether pB169L forms oligomers, we conducted a coimmunoprecipitation (co-IP) assay using HEK293T cells transfected with plasmids expressing the Flag- or EGFP-tagged pB169L. The results showed that the Flag-tagged pB169L interacted with the EGFP-tagged pB169L, indicating that pB169L can form the oligomers (Fig 3C). Consistently, non-denaturing PAGE (native PAGE) analysis demonstrated that pB169L existed in an oligomeric state (Fig 3D). Given that homotypic oligomerization is a hallmark of viroporins and increased membrane permeability is a defining characteristic of these proteins, we investigated the effects of pB169L on membrane permeability. The results showed that all bacteria grew normally in the groups without isopropyl-β-D-thiogalactopyranoside (IPTG) induction, whereas IPTG-induced expression of GST-tagged pB169L (GST-B169L) significantly inhibited bacterial growth compared with GST alone, GST-tagged E301R (GST-E301R), or GST-tagged B169L-ΔTM (GST-B169L-ΔTM), as evidenced by the markedly reduced OD_600nm_ values at all time points (Fig 3E). In addition, Western blot analysis was performed to examine protein expression in the supernatant (S) and pellet (P) fractions. The results showed that GST-B169L was expressed in both the supernatant and pellet fractions after 8 hours of IPTG induction, coinciding with the observed reduction in bacterial growth upon GST-B169L expression (Fig 3F and 3G). Collectively, these results indicate that the expression of full-length pB169L is toxic to bacteria, likely due to its membrane-associated activity, whereas deletion of the transmembrane domain abolishes this inhibitory effect. To further investigate the effect of pB169L on membrane permeability in mammalian cell, we performed the hygromycin B (HygB) permeability assay in HEK293T cells. The results showed that the ectopically expressed pB169L significantly reduced novel protein synthesis in the presence of HygB, whereas the ectopically expressed pE301R had no discernible effect (Fig 3H), indicating that pB169L can significantly increase the membrane permeability of mammalian cells. Taken together, these results suggest that pB169L exhibits the characteristics of a viroporin.

### pB169L functions as a calcium-permeable cation channel

**Fig 4:**
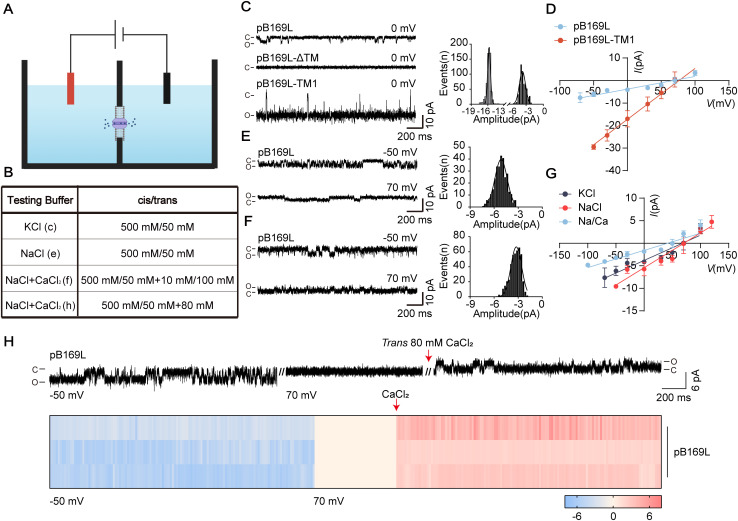
The B169L protein (pB169L) of ASFV serves as calcium-permeable cation channels. **(A)** Schematic diagram of single channel electrophysiological recording by bilayer lipid membrane system. The figure was generated by using the online software BioRender (https://app.biorender.com/). **(B)** Testing buffers used in the single channel recording experiments. **(C)** Single-channel current recording of pB169L, pB169L-ΔTM, and pB169L-TM1 at the indicated potentials and 500 mM: 50 mM KCl solutions. Proteins were added to the cis side. “C” represents closed, and “O” represents open. Current histograms of the traces were shown in the right. **(D)** Linear current-voltage (I-V curve) of pB169L and pB169L-TM1 in 500 mM: 50 mM KCl solution (*n* ≥ 3). **(E)** Single-channel current recording of pB169L at the indicated potentials and asymmetric NaCl solutions. Proteins were added to the cis side. “C” represents closed, and “O” represents open. Current histograms of the traces were shown in the right. **(F)** Single-channel current recording of pB169L at the indicated potentials and asymmetric Na^+^/Ca^2+^ mixed solutions. Proteins were added to the cis side. “C” represents closed, and “O” represents open. Current histograms of the traces were shown in the right. **(G)** I-V curve of pB169L in 500 mM: 50 mM KCl solution, 500 mM: 50 mM NaCl and Na/K solution (*n* ≥ 3). **(H)** Calcium titration measurement of pB169L in 500 mM: 50 mM NaCl solution. Protein was added to the cis side. After stable currents were formed, CaCl_2_ solution was added to the trans side. Heatmap of current signals of the traces was shown below.

To determine whether pB169L functions as a viroporin, we assessed its channel properties using the bilayer lipid membrane (BLM) system (Fig 4A and 4B). When purified pB169L was introduced into an asymmetric potassium chloride solution (500 mM:50 mM), we observed classic single-channel currents (Fig 4C). Based on the structural prediction, we generated two pB169L truncation mutants as controls: one lacking the double TM regions (pB169L-ΔTM) and another retaining only the first TM domain (pB169L-TM1). TM1 was chosen because it constitutes the transmembrane domain that forms hydrophilic side of the pore. As expected, pB169L-ΔTM failed to induce detectable currents under identical recording conditions, whereas pB169L-TM1 displayed single-channel activity. Conductance and open probability analyses demonstrated that pB169L exhibited a single-channel conductance of 56.17 pS, whereas pB169L-TM1, exhibited a markedly higher conductance of ~225.7 pS (Fig 4D). Notably, the preserved channel activity in pB169L-TM1, characterized by increased conductance and elevated open probability (Figs 4D and S4), indicating that the transmembrane domains alone are sufficient to assemble a functional ion channel. Ion selectivity is one of the basic properties of ion channels. Therefore, to assess the ion selectivity of pB169L, we performed electrophysiological recordings in various ionic conditions. In asymmetric NaCl and KCl solutions, pB169L exhibited linear current-voltage (I-V) relationships with reversal potentials of 70.40 and 67.82 mV, respectively (Fig 4E). These values were close to the equilibrium potentials of monovalent cations (K⁺ and Na⁺), indicating that pB169L forms a cation-selective channel. To further evaluate calcium permeability at the single-channel level, we tested pB169L in Na⁺/Ca^2+^ mixed solutions. Typical stepwise currents were observed, and the I-V relationship revealed that adding 10:100 mM (*cis*:*trans)* Ca^2+^ shifted the reversal potential of an asymmetric 50:500 mM Na⁺ solution from 70.40 to 41.56 mV (Fig 4F and 4G), indicating that pB169L is also permeable to Ca^2+^ (PCa^2+^/PNa^+^ = 0.148). To confirm Ca^2+^ conduction through pB169L, we isolated calcium currents. In an asymmetric 50:500 mM (*cis*:*trans*) Na⁺ solution, frequent and stable outward Na⁺ currents indicated the formation of functional pB169L channels in the bilayer lipid membrane (Fig 4H). The shift of the membrane potential to +70 mV abolished Na⁺ currents. Subsequent addition of 80 mM Ca^2+^ induced distinct outward stepwise currents (Fig 4H), further supporting the calcium permeability of pB169L. Collectively, the results indicate pB169L forms a Ca^2+^-permeable cation channel.

### The calcium release-mediated by pB169L is independent of the inositol 1,4,5-trisphosphate receptor (IP3R) channel

Little is known about the biological properties of pB169L. To address this, we examined the transcriptional dynamics of pB169L, as well as its subviral and subcellular localization. A previous study has shown that the *B169L* gene produces two distinct transcripts: a shorter transcript (aa 92–164) expressed during the early phase of infection and the full-length transcript expressed during the late phase of infection [20]. Accordingly, we designed specific quantitative primers to analyze the early and late transcripts separately. The results showed that the shorter *B169L* transcript (aa 92−164) (B169L-1) was transcribed at high levels during the early stages of ASFV infection, although at lower levels than the early-transcribed *CP204L*. In contrast, the transcriptional kinetics of the full-length *B169L* mRNA (B169L-2) closely resembled those of the late gene *B646L* (encoding p72) (S5A Fig). Immunoelectron microscopy revealed that pB169L was localized in the inner envelope of the ASFV virion (S5B Fig). Additionally, laser confocal microscopy showed that pB169L was localized in the endoplasmic reticulum (ER) and Golgi apparatus in ASFV-infected PAMs (S5C and S5D Fig).

Given that the ER is a major Ca^2+^ reservoir and pB169L is localized in the ER, we hypothesized that the channel function of pB169L affects ER calcium homeostasis. Using Fluo-4 to monitor ER calcium stores in HEK293 cells overexpressing pB169L or pB169L-ΔTM, we observed significantly reduced ER Ca^2+^ levels following ionophore-induced ER calcium release compared with controls (S6A and S6B Fig), indicating that pB169L mediates Ca^2+^ efflux from the ER. The data show that the TM regions of pB169L are highly conserved across ASFV strains and play a critical role in pB169L channel function. To determine whether the TM regions are essential for inflammasome activation, HEK293T cells were cotransfected with the plasmid expressing the TM-deleted *B169L* mutant (pB169L-Flag-ΔTM) and the NLRP3-dependent iGLuc reporter as described above. The results indicated that pB169L-ΔTM failed to activate the inflammasome (S6C Fig). To evaluate the functional role of the TM domains of pB169L in NLRP3 activation, HEK293T cells were cotransfected with pFlag-B169L-ΔTM or pFlag-B169L and pMyc-NLRP3 and observed using laser confocal microscopy. The results showed that pB169L-ΔTM was unable to promote NLRP3 aggregation (S6D Fig).

Previously, we have shown that ASFV infection increases intracellular Ca^2+^ levels and that the ER-resident IP3R channel, rather than ryanodine receptor (RyR) channel, is essential for both Ca^2+^ mobilization and ASFV replication [21]. To evaluate the potential role of Ca^2+^ signaling in pB169L-mediated inflammasome activation, we first assessed the cytotoxicity of thapsigargin (Tg) and 2-aminoethoxydiphenyl borate (2-APB) in HEK293T cells. The concentrations of Tg (0.2–10 µM) and 2-APB (5–20 µM) used in this study showed no cytotoxicity to HEK293T cells (S1I and S1J Fig). To determine whether the ASFV pB169L, as a Ca^2+^-permeable cation channel, acts synergistically or redundantly with host Ca^2+^ channels, iGLuc reporter assays were performed with Tg or 2-APB treatment. The results showed that pB169L markedly enhanced IL-1*β*-Gluc activity, whereas the pB169L-ΔTM failed to do so. Importantly, Tg treatment further potentiated pB169L-induced inflammasome activation, while 2-APB failed to suppress pB169L-induced inflammasome activation (S6E and S6F Fig). Given that pB169L is a Ca^2+^-permeable cation channel that robustly activates the inflammasome, we further examined the effect of IP3R inhibition on pB169L-mediated ER Ca^2+^ homeostasis using Ca^2+^ imaging. As shown in S6G and S6H Fig, the pB169L-induced ER Ca^2+^ release was not completely blocked by IP3R inhibition. These results suggest that pB169L-mediated Ca^2+^ conductance is amplified by ER Ca^2+^ independent of IP3R channel. Collectively, pB169L functions as a viroporin and facilitates Ca^2+^ release independent of the host ER IP3R channel.

### The ASFV pB169L mediated-inflammasome activation depends on its characteristic of ion channel

**Fig 5:**
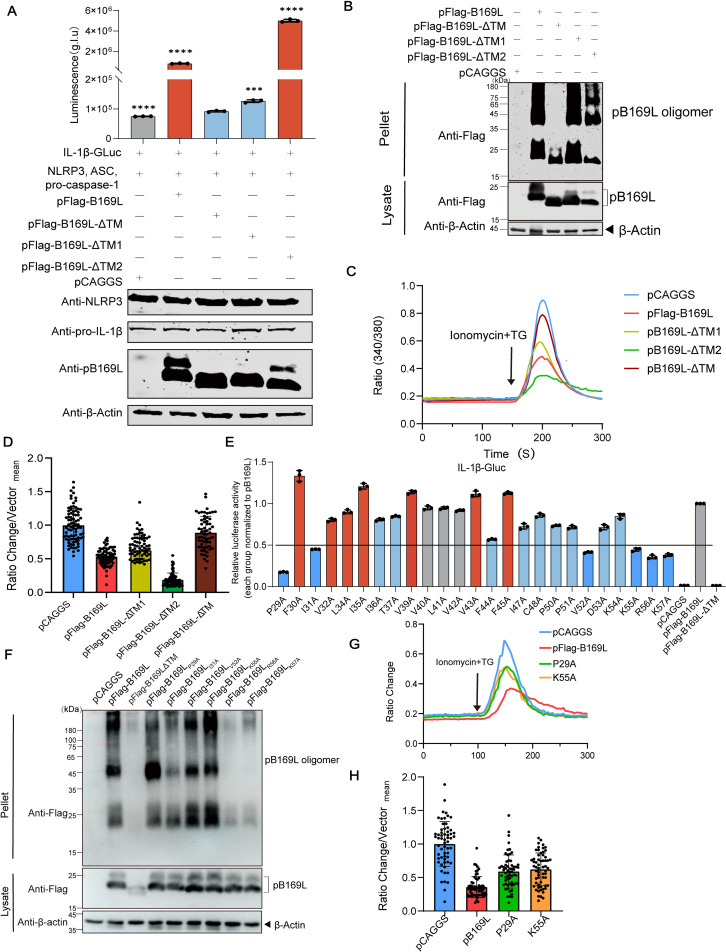
The dominant-negative B169L protein (pB169L) reduces its channel activity and attenuates inflammasome activation. **(A)** iGLuc luciferase activity analysis of pB169L domains. HEK293T cells were cotransfected with pFlag-B169L, pFlag-B169L-ΔTM, -ΔTM1, or -ΔTM2 in the presence of the iGLuc-based NLRP3 inflammasome system, and the supernatants were collected to determine luciferase activity at 24 hours posttransfection (hpt). **(B)** Oligomerization analysis of pB169L domains. HEK293T cells were transfected with 2 µg of the indicated plasmids for 24 hours, followed by DSS cross-linking assay using 4 µM DSS (final concentration: 4 μM) at 4°C for 30 minutes. Protein oligomerization was analyzed by Western blotting. **(C)** Calcium imaging analysis of pB169L domains. Representative average traces of Ca^2+^ image of HEK293 cells transfected with pCAGGS, pFlag-B169L, pFlag-B169L-ΔTM1, pFlag-B169L-ΔTM2, or pFlag-B169L-ΔTM. HEK293 cells were transfected with indicated plasmids. Fura-2 AM was used to monitor ER calcium storage. Ionomycin and Thapsigargin were added at the time indicated by the arrow. **(D)** Scatter plot of ionomycin and thapsigargin (Tg) induced ratio change of cells transfected with pCAGGS, pFlag-B169L, pFlag-B169L-ΔTM1, pFlagB169L-ΔTM2, or pFlag-B169L-ΔTM (*n* > 40). **(E)** iGLuc luciferase activity analysis of pB169L mutations. Each residue of pB169L-TM1 (aa 29-57) was substituted with alanine and the influences of each mutation on the pB169L-induced NLRP3 inflammation was examined as iGLuc-luciferase activity. **(F)** Oligomerization analysis of pB169L mutations. HEK293T cells were transfected with different point mutated plasmids. At 24 hpt, the cells were incubated with 4 μM DSS at 4°C for 30 minutes. The cells were incubated with 50 µL of 2 × protein loading buffer. The oligomerization was evaluated by Western blotting analysis. **(G)** Calcium imaging analysis of pB169L mutations. Representative average traces of calcium imaging of HEK293 cells transfected with different point mutated plasmids. HEK293 cells were transfected with different point mutated plasmids. At 24 hpt, the cells were loaded with Fura-2 to monitor ER calcium storage. Ionomycin and Tg were added at the time indicated by the arrow. **(H)** Scatter plot of ionomycin induced ratio change of HEK293 cells transfected with different point mutated plasmids. Each point represents a single cell. All the data were analyzed using the Student’s *t* test. **, *P* < 0.01, ***, *P* < 0.001; ns, not significant, *P* ≥ 0.05.

It has been shown that pB169L fragment spanning aa 23–57—which includes the first transmembrane domain (TM1)—possesses pore-forming activity [18]. Our results further demonstrate that both the TM1 region and the full-length pB169L are capable of forming Ca^2+^-permeable cation channel (Fig 4). To verify the association between the pore-forming function of pB169L and the proinflammatory response, we generated a series of mutants lacking either TM1 (pB169L-ΔTM1) or TM2 (pB169L-ΔTM2) (S7A Fig). Subsequently, the mutant plasmids were co-transfected with the iGLuc reporter system into HEK293T cells. The pB169L-ΔTM1 markedly reduced inflammasome activation compared with pB169L. Interestingly, the pB169L-ΔTM2 significantly enhanced the inflammasome activation, exceeding the level induced by pB169L (Fig 5A). Furthermore, DSS cross-linking assays revealed that pB169L proteins formed oligomers, and both pB169L-ΔTM1 and pB169L-ΔTM2 could be formed pronounced oligomerization pattern (Fig 5B). Consistent with inflammasome activation results, ER calcium content changes tested by Ca^2+^ imaging showed a similar tendency. ER calcium content of pB169L-ΔTM1 expressing cells was higher than pB169L expressing cells, and pB169L-ΔTM2 led to a lower ER Ca^2+^ content. (Figs 5C and S7B). These results indicate that TM1 positively regulates pB169L-induced inflammasome activation and Ca^2+^ signaling, highlighting differential contributions of pB169L-ΔTM in modulating proinflammatory responses, indicating a direct association between pB169L-induced inflammation and the viroporin function.

As shown in S3 Fig, the residues 29–48 within the TM1 region of the channel interior. Given the critical role of TM1 in inflammasome activation, a series of mutant pFlag-B169L plasmids containing the TM1 region and its adjacent sequence (aa 29–57) were constructed and transfected HEK293T cells together with the NLRP3-dependent iGLuc reporter. The protein expression levels among the mutants remained consistent (S7C Fig). In comparison with the pB169L group, the mutations at residues P29, I31, V52, K55, R56, and K57 exhibited highly significant inhibitory effects (with a fold change of > 2) (Fig 5D). To investigate the association between ion channel activities and the key aa affecting the pB169L-induced NLRP3 inflammasome activation, the oligomerization of the pB169L mutations was determined by DSS cross-linking assay. The K57A mutation significantly impaired pB169L oligomerization (Fig 5E), indicating that the K57A mutation impaired the inflammasome activation through disrupting pB169L oligomerization. The rest mutants were further examined by calcium imaging. As shown in Figs 5F and S7D, P29A and K55A mutations partially restored the reduced ER Ca^2+^ storage in cells compared with that of pB169L, indicating that P29 and K55 may be the key amino acids of pB169L channel function. Furthermore, other mutations did not restore reduced ER calcium stores in the cells (S7D Fig). In summary, P29 and K55 are involved in the pB169L-induced NLRP3 inflammasome activation by regulating the ER Ca^2+^ efflux. Overall, these results highlight the mechanistic link between pB169L oligomerization, ER Ca^2+^ dysregulation, and inflammasome activation, providing new insights into the viroporin function of the ASFV pB169L.

### The ASFV pB169L is essential in the ASFV-induced proinflammatory responses

**Fig 6:**
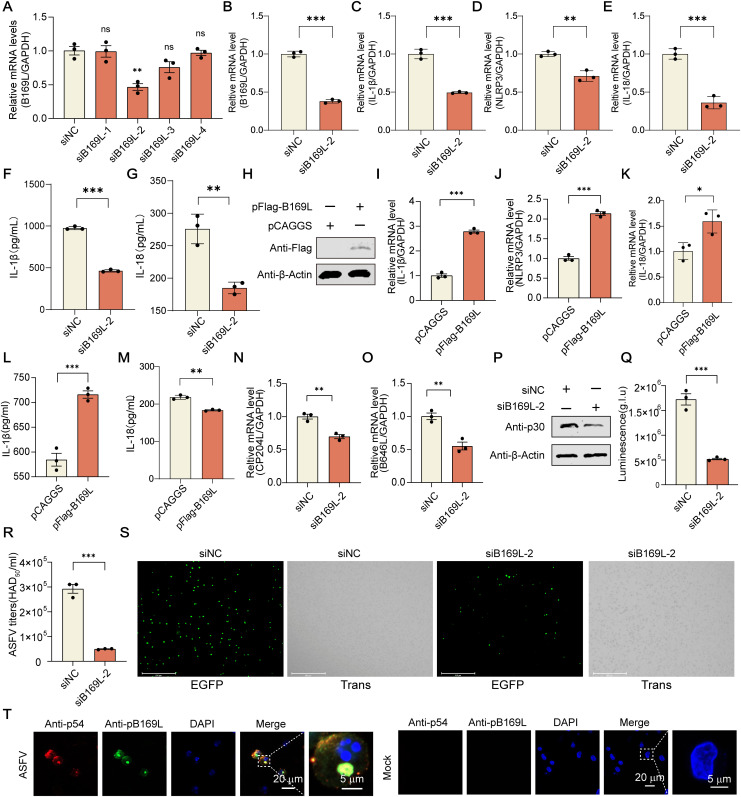
The B169L protein (pB169L) is essential for IL-1*β* production induced by ASFV infection. **(A)** Effectiveness of siRNAs targeting the ASFV *B169L* gene. PAMs were transfected with siB169Ls and infected with ASFV (multiplicity of infection (MOI) = 0.1) at 8 hours posttransfection (hpt). At 2 hours postinfection (hpi), the supernatants were discarded and replaced with fresh medium. The transcription level of *B169L* was quantified by reverse transcription-quantitative PCR (RT-qPCR) at 24 hpi. **(B–G)** Effects of ASFV *B169L* gene knockdown on proinflammatory cytokines. PAMs (10^6^ cells/well) were transfected with siRNA-2. At 8 hpt, the cells were infected with ASFV (MOI = 0.1). The transcription levels of *B169L, IL-1*β, *NLRP3,* and *IL-18* were quantified by RT-qPCR at 24 hpi. The supernatants were collected and the secretion of IL-1*β* (F) and IL-18 (G) was tested by enzyme-linked immunosorbent assay (ELISA). **(H–M)** Impact of overexpression pB169L in WSL cells on proinflammatory cytokines. WSL cells were transfected with pFlag-B169L or pCAGGS. At 24 hpt, the transcriptional of *IL-1β*, and NLRP3 were analyzed by RT-qPCR. The supernatants were detected by a porcine IL-1*β*/IL-18 ELISA kit. **(N–P)** Effects of ASFV *B169L* gene knockdown on ASFV replication. PAMs were transfected with siB169L-2, followed by infection with ASFV (MOI = 0.1) at 8 hpt. At 24 hpi, the transcription of *CP204L* (encoding p30) and *B646L* (encoding p72) and the expression of p30 were analyzed by RT-qPCR or Western blotting. **(Q–S)** Effects of ASFV *B169L* gene knockdown on ASFV replication. PAMs were transfected with siB169L-2, followed by infection with rASFV-EGFP/Gluc (MOI = 0.1) at 8 hpt. At 24 hpi, the luciferase values, viral titers, and fluorescence signals of EGFP were analyzed. **(T)** Subviral localization of pB169L. PAMs were infected with ASFV-WT at an MOI of 1. At 24 hpi, the fluorescent signals were analyzed by laser confocal microscopy using rabbit anti-pB169L PAb, mouse anti-p54 PAb, and DAPI. Scale bar, 20 µm. All the data were analyzed using the Student’s *t* test. **, *P* *<* 0.01, ***, *P* *<* 0.001; ns, not significant, *P* ≥ 0.05.

To investigate the effects of pB169L on IL-1*β* secretion in the context of ASFV infection, PAMs were transfected with siRNAs targeting the *B169L* gene and subsequently infected with ASFV. The results demonstrated that 200 nM siB169L-2 effectively inhibited *B169L* mRNA transcription (Fig 6A), without inducing cytotoxicity in PAMs (S1K Fig). Moreover, *B169L* knockdown led to a reduced the transcription of *IL*-*1β*, *IL-18*, and *NLRP3* (Fig 6B−6E), as well as a significant decrease in the secretion of IL-1*β* and IL-18 in the supernatants of PAMs infected with various MOIs of ASFV (Figs 6F, 6G, and S8A**−**S8F Fig). To further validate the effects of pB169L on the proinflammatory cytokine production, WSL cells transfected with pFlag-B169L exhibited the increased transcription of intracellular *IL-1β*, *IL-18*, and *NLRP3* (Fig 6H**−**6K), as well as exhibited enhanced the secretion of IL-1*β* and IL-18 in the supernatants (Fig 6L and 6M). Furthermore, HEK293T cells co-transfected with pFlag-B169L and the iGLuc reporter system revealed that pB169L was capable of cleaving GSDMD to generate the N-terminal fragment (GSDMD-N) (S8G Fig). Collectively, these data suggest that pB169L promotes the activation of the NLRP3-dependent inflammasome, thereby enhancing IL-1*β* and IL-18 secretion and contributing to the proinflammatory response during ASFV infection.

Generally, inflammatory responses not only mediate host immune defense but also influence viral replication. Accumulating evidence suggests that virus-induced inflammatory signaling can create a favorable intracellular environment for viral replication or dissemination. To determine whether our findings are relevant in the context of viral infection, we next assessed the role of pB169L during ASFV infection of porcine macrophages. We demonstrated that pB169L is a structural protein localized in the inner envelope of virions and involved in proinflammatory responses. Therefore, we examined the involvement of pB169L in ASFV replication. PAMs were transfected with siB169L-2 followed by ASFV infection. The results showed that silencing *B169L* significantly reduced the mRNA levels of *B646L* (encoding the viral structural protein p72) and *CP204L* (encoding the viral structural protein p30) (Fig 6N and 6O). The expression of p30 was also reduced (Fig 6P). To further assess the effect of *B169L* knockdown on viral replication, PAMs were transfected with siB169L-2 and then infected with rASFV-Gluc/EGFP. Compared with the siNC control, silencing *B169L* resulted in a significant reduction in Gluc activities (Fig 6Q), viral titer in the supernatants (Fig 6R), and the EGFP fluorescence in the cells (Fig 6S). In addition, confocal microscopy revealed that pB169L and p54 colocalized in the viral factories of the ASFV-infected PAMs (Fig 6T). In summary, these findings underscore the essential role of ASFV pB169L in regulating the proinflammatory response and promoting viral replication, highlighting its dual contribution to both host inflammation and viral pathogenesis.

## Discussion

The ASFV genome encodes over 160 proteins, several of which have been shown to antagonize host innate immune pathways, particularly the interferon (IFN) signaling and proinflammatory suppression. More specifically, viral proteins, such as pDP96R [22], pE120R [23], pMGF505-7R [24], and pI267L [25], have been identified as antagonists of IFN responses in the IFN pathway. In terms of antagonizing the proinflammatory responses, several viral proteins, including pL83L [26], pA238L [27], pMGF300-4L [28], pMGF505-2R [29], pMGF505-7R [30], pA137R [31], and pH240R [32], have been shown to inhibit proinflammatory responses. A recent study has shown that the I177L protein promotes inflammatory responses, whereas deletion of this gene results in attenuated proinflammatory responses [33]. Although numerous ASFV proteins have been reported to antagonize host innate immunity, relatively few are known to actively promote proinflammatory signaling. Our study identifies pB169L as such a factor. In this study, we provide the first direct evidence that the ASFV structural protein pB169L forms a channel to drive virus-induced proinflammatory responses.

ASFV infection induces a cytokine storm in domestic pigs, characterized by elevated proinflammatory cytokines, such as IL-1*β* [3]. A previous study indicates that the necrosome is mediated by ZBP1-RIPK3-MLKL axis in ASFV-induced proinflammatory responses [34]. Ion imbalance is increasingly recognized as a pivotal trigger of inflammatory cell death and cytokine production [35–37]. Consistently, our data show that pB169L, through its viroporin activity, disrupts intracellular calcium homeostasis, leading to NLRP3 inflammasome activation and IL-1*β* secretion. pB169L induced a robust proinflammatory response and the ability to induce IL-1*β* production. We presume that pB169L is an important inflammatory mediator in ASFV pathogenesis. Future studies will be conducted to construct mutant viruses to investigate the correlation between pB169L-induced proinflammatory responses and pathogenicity in pigs.

Calcium ions, as essential second messengers, play a critical role in regulating the cell cycle. Recently, we have shown that ASFV modulates cellular calcium channels IP3R to promote calcium release and thereby facilitating viral replication [21]. In this study, we demonstrate that ASFV encodes a viroporin capable of forming Ca^2+^-permeable channels, enabling the virus to manipulate calcium flux. Therefore, whether ASFV—beyond hijacking host Ca^2+^ channels for viral replication—also contributes to Ca^2+^ channel formation via its own encoded proteins to facilitate viral replication is an important area for future exploration.

**Fig 7:**
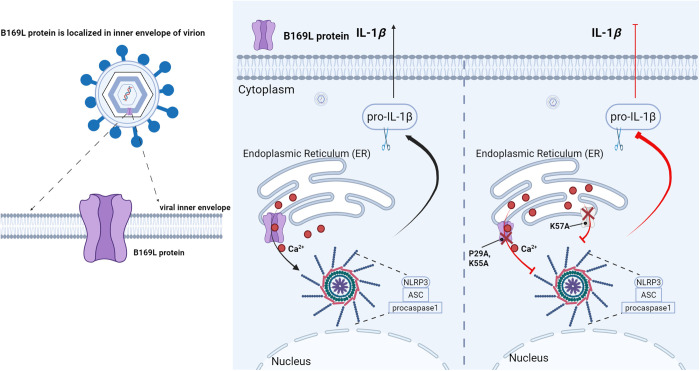
Schematic diagram of the ASFV pB169L-induced activation of NLRP3 inflammasome. pB169L localizes to the ER to promote calcium release and activate the NLRP3 inflammasome. The P29A and K55A mutation reduces calcium efflux and weakens NLRP3 inflammasome activation, while the K57A mutation disrupts B169L oligomerization and impairs NLRP3 inflammasome activation. The figure was generated by using the online software BioRender (https://app.biorender.com/).

In this study, we demonstrated that pB169L exhibits typical features of viroporins. Structural modeling predicted that pB169L assembles into a pentameric channel with high confidence, and electrophysiological studies confirmed its function as a Ca^2+^-permeable channel, and that its overexpression markedly increases intracellular calcium levels and proinflammatory responses. However, the removal of the transmembrane domains completely loses such functions. Mutation analysis indicated that P29, K55, and K57 residues are critical for the inflammatory activation, respectively. Notably, mutations at P29 and K55 impaired calcium flux and attenuated inflammasome activation without affecting oligomerization, while mutation at K57 abolished both oligomerization and downstream inflammatory signaling (Fig 7). These results reveal that multiple residues cooperatively contribute to pB169L-mediated inflammasome activation through distinct mechanisms involving both oligomerization and ion conductance. Lysine is a positively charged residue which does not usually function as a canonical selectivity filter element. Common calcium selectivity filter elements are acidic residues (E/D). Our data showed that the K55A mutant partially restores ER Ca^2+^ content and reduced the inflammatory. K55 may not form a classical selectivity filter, but rather modulate local electrostatics or pore conformation, thereby indirectly influencing Ca^2+^ leak. A positively charged lysine at this position may reduce the ability of nearby acidic residues to coordinate Ca^2+^, facilitating constitutive Ca^2+^ efflux. Substitution with alanine removes this charge, decreases leakiness, and allows ER Ca^2+^ stores to recover. Similar phenomena have been described for other cation channels, where single positively charged residues can reshape local electrostatics and alter ion selectivity. These findings underscore the essential role of pB169L channel activity in mediating ASFV-triggered inflammation. Consistent with its viroporin nature, pB169L localizes predominantly to the ER, a major intracellular calcium reservoir. The ER localization of pB169L and its ability to deplete ER calcium stores provide compelling evidence that ER calcium efflux underlies inflammasome activation in ASFV-infected cells. Pharmacological blockade of calcium flux with BAPTA-AM effectively suppressed pB169L-mediated inflammasome activation, ASC speck formation, and NLRP3 oligomerization, further validating the central role of calcium disturbance in this process. Taken together, these data indicate that the transmembrane domain of pB169L is required for inflammasome activation and suggest that pB169L function is linked to calcium homeostasis, which can be modulated by ER calcium release.

Although viroporins from other viruses, such as the HCV p7 protein and EMCV 2B protein, have been implicated in modulating ion homeostasis and inflammasome activation, the channel activities and key sites of most viroporins have not been directly confirmed. Concretely speaking, their association with ions has only been demonstrated through indirect assays, which are more often through inhibitor experiments. For example, the duck hepatitis A virus 1 (DHAV-1) 2B protein disrupts ion and organelle homeostasis to promote proinflammatory responses [38]. The EMCV 2B protein enhances Ca^2+^ flux from intracellular stores to the cytosol, facilitating NLRP3 activation and IL-1*β* production [17]. Furthermore, the rhinovirus 2B protein forms oligomers that create pores in the membranes of the ER and Golgi complex, reducing Ca^2+^ and H^+^ levels in the lumen of these organelles and leading to increased IL-1 production [39]. It is worth noting that there are also few studies validating channel properties using biochemistry methods. For instance, the respiratory syncytial virus (RSV) SH protein accumulates in the Golgi network within the lipid raft structure, forming ion channels selective for monovalent cations (Na^+^ and K^+^), which trigger the translocation of NLRP3 from the cytoplasm to the Golgi network and its subsequent activation [40]. Additionally, the IAV M2 protein enables H^+^ export from the acidified Golgi, providing a secondary signal for NLRP3 activation that affects ROS production and K^+^ efflux to promote IL-1*β* production [16]. Unlike previous studies that inferred viroporin activity mainly through inhibitor assays, our work provides direct electrophysiological evidence for ASFV pB169L channel activity. Our study directly establishes a connection between the ion channel activity of an ASFV protein and inflammasome activation through biochemical experiments involving mutations in different structural domains. Importantly, we show that silencing *B169L* not only reduces IL-1*β* production but also impairs ASFV replication, underscoring its essential role in ASFV pathogenesis. These dual roles make pB169L an attractive target for antiviral strategies and rational vaccine design.

Our study complements and extends recent work by Gladue et al. [18], who demonstrated that the hairpin transmembrane domain of pB169L exhibits viroporin-like activity but did not assess the calcium permeability of the full-length protein or its involvement in inflammasome activation. Beyond pB169L, ASFV encodes additional proteins with potential viroporin-like activity. For example, pB117L has been reported as a membrane-active protein with potential viroporin-like activity that may perturb cellular ion balance [18,21]. In addition, ASFV entry is mediated by a multi-subunit entry–fusion complex (EFC), two conserved components of which, pE199L and pE248R, are shared with other NCLDVs [41–44]. In the present study, both pE199L and pE248R promoted IL-1*β* secretion (Fig 1), indicating that, in addition to their essential roles in membrane fusion and viral entry, these proteins may also contribute to proinflammatory responses. Collectively, these observations suggest that ASFV may encode multiple viroporin-like proteins that could act at distinct stages of infection to modulate calcium signaling, inflammasome activation, and proinflammatory outcomes. In this study, we demonstrated for the first time that full-length pB169L forms Ca^2+^-permeable cation channels and that the TM1 region is critical for NLRP3 inflammasome activation and IL-1*β* secretion. These observations suggest that pB169L may serve dual functions—acting both as a structural component essential for viral replication and as a viroporin that inadvertently drives host inflammatory responses.

Our findings demonstrate that pB169L is a cation-selective channel protein located in the inner envelope of virion and is crucial for the viral replication. Therefore, despite multiple attempts using the homologous recombination method described previously [45], we failed to generate a *B169L*-deficient ASFV. It is likely that a conditionally expressed mutant virus is an effective alternative approach. Future research will construct such a mutant virus to test the functional role of pB169L in ASFV infection *in vitro*. Furthermore, the involvement of pB169L in virulence should be validated in pigs using the conditionally expressed mutant virus in future studies.

In conclusion, our findings reveal a novel mechanism by which the ASFV pB169L forms calcium-permeable cation channels to trigger NLRP3 inflammasome activation through disrupting intracellular Ca^2+^ homeostasis, driving proinflammatory responses during infection. Given the essential role of B169L, targeting pB169L may represent a promising therapeutic approach to mitigate ASFV-induced inflammation and disease severity, offering valuable insights for the development of antiviral interventions and gene-deletion vaccines against ASF.

## Materials and methods

### Ethics statements

All experiments of live ASFV had been approved by Animal Biosafety Level 3 (ABSL-3) facilities in the Harbin Veterinary Research Institute (HVRI), the Chinese Academy of Agricultural Sciences (approval number: HVRISQ-24–13).

### Biosafety statement and facilities

All experiments with live ASFV were conducted in Animal Biosafety Level 3 (ABSL-3) facilities at Harbin Veterinary Research Institute (HVRI), the Chinese Academy of Agricultural Sciences.

### Cells and antibodies

HEK293T and HEK293 cells were cultured in high-glucose DMEM supplemented with 10% heat-inactivated FBS and 1% antibiotics (10,000 IU/mL penicillin, 10,000 µg/mL streptomycin). PAMs isolated from the lung lavage fluid of 28-day-old healthy specific-pathogen-free (SPF) piglets, WSL cells (the susceptible cells for ASFV) gifted by Prof. Jun Han at China Agricultural University were cultured in RPMI-1640 medium supplemented with 10% FBS and 2% antibiotics. HEK293T cells, WSL cells, and PAMs were cultured at 37°C with 5% CO_2_.

Rabbit anti-*β*-actin polyclonal antibodies (PAbs) (catalog no. AE005) were purchased from ABclonal. Rabbit anti-Flag (catalog no. F7425), mouse anti-Flag (catalog no. F1804), and mouse-anti-Myc (catalog no. M4439) PAbs were purchased from Sigma-Aldrich. Alexa Fluor 488-conjugated donkey anti-mouse IgG (H + L) (catalog no. A11029), anti-rabbit IgG (H + L) (A11008), Alexa Fluor 633-conjugated goat anti-rabbit IgG (H + L) (catalog no. A11029), IRDye 800CW goat anti-rabbit IgG (H + L) (catalog no. 926-32211) and goat anti-mouse IgG (H + L) (catalog no. 926-32212) antibodies were purchased from LI-COR Biosciences. Anti-Flag M2 magnetic beads (catalog no. M8823) were purchased from Sigma-Aldrich. Rabbit anti-p30 PAbs and mouse anti-p54 were prepared as described previously [46]. 4’,6-Diamidino-2-phenylindole (DAPI) (catalog no. C006) was purchased from Solarbio.

### Plasmid construction and cell transfection

The pEGFP-B169L, pFlag-B169L and pFlag-B169L-ΔTM plasmids were synthesized by the Beijing Genomics. A series of plasmid encoding site directed *B169L* mutants for alanine scanning were synthesized and sequenced by Genesoul Technology Institute. HEK293T cells were transfected with these expression plasmids using the X-tremeGENE HP DNA transfection reagent (catalog no. 06366236001; Roche) according to the manufacturer’s protocols.

### Virus infection and titration

The ASFV HLJ/2018 strain [47] (GenBank accession no. MK333180.1) and the reporter virus rASFV-Gluc/EGFP [48] were propagated in PAMs. Following infection, the virus-containing supernatants were collected and centrifuged at 500 × *g* for 5 minutes to eliminate cell debris, and then aliquots were stored at –80°C for subsequent analysis.

### Luciferase reporter assay

The NLRP3 inflammasome reporter assay was performed as described previously [32,49]. Briefly, HEK293T cells were cotransfected with pB169L-expressing plasmid pFlag-B169L and the NLRP3 inflammasome-dependent iGLuc reporter (60 ng/well for pFlag-NLRP3, 10 ng/well for pFlag-pro-Caspase-1, 10 ng/well for pFlag-ASC, and 300 ng/well for pFlag-iGluc), and then transfected with a plasmid expressing the corresponding protein for 24 hours. The cell supernatants (20 µL/well) were collected, and the mature IL-1*β-*Gluc was analyzed by using a Pierce *Gaussia* luciferase flash assay kit (catalog no. 16159; Thermo Fisher Scientific) according to the manufacturer’s instructions. The intracellular proteins were determined by Western blotting analysis.

To investigate the effects of pB169L on the virus production, PAMs were transfected with siIB169-2 and infected with rASFV-Gluc/EGFP at 8 hours posttransfection (hpt). At 2 hpi, the cells were washed with PBS and incubated with fresh medium. At 24 hpi, the supernatants (20 μL) were collected and added to 96-well solid black bottom plates (Biosharp, BS-MP-96B-CL). The Gluc substrate (30 μL) was added to each well. The Gluc activities in the supernatants of each well were then measured using the EnVision HTS microplate reader (PerkinElmer).

### Purification of the pB169L and the TM-deleted pB169L (pB169L-ΔTM)

The full-length or TM-deleted *B169L* gene fused with the IL-2 signal peptide at its 5’-terminus and the His-strep tag at the 3’-terminus was cloned into the pCAGGS vector to generate the plasmids pCA-SP-B169L-HS and pCA-SP-B169L(ΔTM)-HS, respectively. HEK293S suspension cells were transduced with the pCA-SP-B169L-HS or pCA-SP-B169L(ΔTM)-HS (500 µg) using PEI Max. The cells were cultured in suspension at 37°C and shaken at 110 rpm for 3 days. The cell suspension was expanded, and the cell supernatants were collected. The supernatants were filtered through a 0.45-µm filter, and resin was added at a ratio of 2 mL of Ni^2+^ resin per 100 mL of cell culture supernatants. The mixture was passed through the resin column 3–4 times. The resin was then washed with 5 column bed volumes of 20 mM imidazole buffer, 50 mM imidazole buffer, 100 mM imidazole buffer, and 200 mM imidazole buffer. Finally, the protein was concentrated using an ultrafiltration tube to obtain purified pB169L or pB169L-ΔTM. Protein purity was assessed by SDS-PAGE, followed by Coomassie brilliant blue staining and Western blotting analysis.

### Prediction of the viroporin motifs in pB169L

The pB169L TM domain was predicted using the DeepTMHMM-1.0 (https://services.healthtech.dtu.dk/services/DeepTMHMM-1.0/). The secondary structure of pB169L was predicted using the PSIPRED (http://bioinf.cs.ucl.ac.uk/introduction/) (Buchan and Jones, 2019).

### Single-channel recording

Purified pB169L, pB169L-ΔTM, and pB169L-TM1 proteins were incorporated into bilayer lipid membrane. All lipids were obtained from Avanti Polar Lipids (USA). The bilayers were composed of phosphatidylcholine and phosphatidylserine at a 3:2 ratio. The experimental setup consisted of a *cis* compartment and a trans compartment. The *cis* solution typically contained 500 mM KCl and 5 mM HEPES (pH 6.35), while the *trans* solution comprised 50 mM KCl and 5 mM HEPES (pH 6.35). Purified proteins were added to the *cis* side, and their incorporation into the bilayer lipid membrane was facilitated by electrochemical gradients and mechanical stirring. Recordings were performed using a Warner BC-535 bilayer clamp amplifier (Warner Instruments, USA) in a voltage-clamp mode. Current signals were digitized with the pClamp 10.2 software (Molecular Devices, USA). Channel conductance and opening times were calculated by fitting data using Gaussian distribution functions or single/double exponential equations. Signals with opening times < 0.5 to 1.5 ms were excluded from statistical analyses. Reversal potentials in single-ion or multi-ion solutions were calculated using the Nernst or Goldman-Hodgkin-Katz equations, as appropriate.

### Measurement of calcium stores

HEK293 cells were seeded onto poly-L-lysine-coated 96-well plates and cultured overnight to allow attachment. After overnight incubation, cells were transfected with either pFlag-B169L or pCAGGS. At 6 hpt, the medium was replaced, and cells were incubated for an additional 24 hours. The culture medium was then removed, and cells were incubated at 37°C for 1 hour with 2.5 μM Fluo-4 solution in the calcium-free buffer. After incubation, unbound dye was removed by washing once with the calcium-containing buffer, followed by three washes with the calcium-free buffer. Fluorescence measurements were performed using the FDSS/µCell system (Hamamatsu Photonics, Japan) with excitation/emission wavelengths of 480 or 540 nm. During the assay, ionomycin (catalog no. HY-13434; MedChemExpress) was added to each well at a final concentration of 2 μM to induce calcium release from the ER into the cytoplasm. The calcium-free buffer contained 140 mM NaCl, 5 mM KCl, 1 mM MgCl_2_, 10 mM glucose, 2 mM probenecid, and 10 mM HEPES (pH adjusted to 7.2 with 2 M NaOH). The calcium-containing buffer was prepared by adding CaCl_2_ to the calcium-free buffer with a final concentration of 2 mM.

### Inhibitor treatment assay

HEK293T cells were seeded in 24-well plates and cotransfected with the plasmids pFlag-NLRP3 (60 ng/well), pFlag-ASC (10 ng/well), pFlag-pro-caspase-1 (10 ng/well), pFlag-pro-IL-1*β*-Gluc (pFlag-iGluc) (300 ng/well), and pFlag-B169L (1 μg/well). At 24 hpt, the cells were incubated with different inhibitors of ion channels, including K^+^ (10 μM glibenclamide and 1 mM 4-aminopyridine), Ca^2+^ (25 μM BAPTA-AM, 0.6 mM EGTA, and 50 μM verapamil), Na^+^/H^+^ [10 μM 5-(N, N-hexamethylene)-amiloride and 15 μM ethylisopropylamiloride] for 12 hours. The cell supernatants (20 μL/well) were collected for the examination of iGLuc activities using a Pierce *Gaussia* luciferase flash assay kit (catalog no. 16159; Thermo Scientific) according to the manufacturer’s instructions.

### Transmission electron microscopy (TEM)

To determine the localization of pB169L on ASFV virions, PAMs were infected with ASFV-WT at a multiplicity of infection (MOI) of 3, and mock-treated PAMs were included as a control. At 24 hours postinfection (hpi), the cells were fixed with 2% glutaraldehyde in PBS for 2 hours at 4°C and embedded in epoxy. After polymerization, 80-nm-thick (ultrathin) sections were prepared and stained with uranyl acetate and lead citrate according to the manufacturers’ protocols. The samples were then analyzed on an H-7650 instrument (Hitachi, Tokyo, Japan) operated at 80 kV.

### Laser confocal microscopy

To analyze the formation of NLRP3 aggregates, pMyc-NLRP3 was cotransfected with pFlag-B169L or pCAGGS into HEK293T cells for 24 hours. For the ASC speck formation assay, HEK293T cells were transfected with pFlag-B169L or pCAGGS and pMyc-ASC for 24 hours to confirm the specific formation of specks. At 24 hpt, the cells were fixed with 4% paraformaldehyde (catalog no. BL539A; Biosharp) for 30 minutes, washed with PBS for three times, and then permeabilized or not with 0.15% Triton X-100 (catalog no. 1139100; BioFroxx) for 15 minutes at room temperature. The cells were imaged using a Zeiss LSM 880 laser-scanning confocal microscope after incubation with the relevant primary and secondary antibodies.

### Detection of intracellular Ca^2+^ concentrations

The cytosolic Ca^2+^ was examined using a Fluo-4 Flux analysis kit (catalog no. S1061S; Beyotime). Briefly, HEK293T cells were transfected with pFlag-B169L (0, 50, 100, 150, or 200 ng). At 24 hpt, the cells were treated with Fluo-4 AM for 30 minutes at 37°C. The fluorescence was measured using a multifunctional microplate reader (Thermo Fisher Scientific) at an emission wavelength of 485 nm and an excitation wavelength of 535 nm.

### Membrane permeabilization assay

HygB is widely employed to assess changes in membrane permeability due to its unique properties: it inhibits cellular protein synthesis while remaining impermeable to cells over a short period of time [50]. HEK293T cells were transfected with the indicated plasmids for 24 hours and then treated with the indicated concentration of HygB for 2 hours at 37°C. Next, *de novo* synthesized proteins were labeled with 5 µg/mL puromycin (Beyotime, China) in the presence or absence of HygB for 25 minutes [51]. The synthesis of novel cellular proteins was examined by Western blotting analysis using the anti-puromycin monoclonal antibody (MAb).

### Bacterial lysis assay and Western blotting analysis

To assess whether pB169L enhances the membrane permeability, we performed the *Escherichia coli* lysis assay by examining the optical density at 600 nm (OD_600nm_) of *E. coli* as described previously [52]. The *E301R*, *B169L*, and *B169L(*Δ*TM*) genes were cloned into the pGEX-6P-1 vector to generate pGEX-E301R, pGEX-B169L, and pGEX-B169L(ΔTM), respectively. *E. coli* BL21(DE3) pLysS cells were transformed with each of the above plasmids and cultured overnight in Luria-Bertani (LB) medium supplemented with 100 µg/mL ampicillin at 37°C. Isopropyl-*β*-D-thiogalactopyranoside (IPTG) was added to the cells to induce protein expression when the optical density at 600 nm (OD_600nm_) reached 0.8. The OD_600nm_ values were recorded in triplicates over a time course of 1–7 hours postinduction. The bacterial culture was centrifuged, after which the supernatant was discarded, and the bacterial was resuspended in ice-cold PBS. The bacterial pellet was disrupted by ultrasonication using an Ultrasonic Crusher under standard conditions in an ice-water bath. Following disruption, the lysate was centrifuged to separate the supernatant (S) and pellet (P), which were subsequently subjected to Western blotting analysis.

### Co-IP assay and Western blotting analysis

For the co-IP assay, HEK293T cells transfected with the indicated plasmids were harvested and lysed with NP-40 lysis buffer for 30 minutes at 4°C with gentle shaking. The cell lysates were centrifuged at 12,000 × *g* for 10 minutes at 4°C, and the supernatants were subsequently incubated with anti-*c*-Myc magnetic beads (catalog no. HY-K0206; MCE), anti-Flag M2 magnetic beads (catalog no. M8823; Sigma-Aldrich) or anti-protein A/G magnetic beads (catalog no. HY-K0202; MCE) at 4°C overnight. The beads were then washed five times with PBS and lysed in SDS loading buffer for further analysis. The lysates were separated by 12.5% SDS-PAGE and transferred to nitrocellulose membranes (Bio-Rad, Hercules, CA, USA) for the Western blotting analysis.

### Dioctanoyl di-succinimidyl ester (DSS) cross-linking assay

To detect the effects of pB169L on the oligomerization of NLRP3 or ASC, HEK293T cells were transfected the corresponding plasmids. At 24 hpt, cells were lysed in NP40 buffer on ice for 10 minutes, and lysates were centrifuged at 5000 rpm for 10 minutes. The supernatant was mixed with 5 × loading buffer and heated at 98°C for 7 minutes. The pellets were washed three times with PBS, centrifuged, incubated with 2 mM DSS cross-linker (final 4 μM) for 30 minutes, and resuspended in 1 × loading buffer before heating at 98°C for 2 minutes. Lysate and pellet samples were separated by 12.5% SDS-PAGE and transferred to nitrocellulose membranes (Bio-Rad, Hercules, CA, USA) for the Western blotting analysis.

### RNA interference assay

The siRNAs targeting *B169L* (siB169L) were designed and synthesized by GenePharma. PAMs were transfected with 200 nM siB169L using X-tremeGENE siRNA transfection reagent (catalog no. 04476115001; Roche) for 8 hours and then infected with the ASFV HLJ/2018 strain (MOI = 0.1). At 2 hpi, fresh RPMI-1640 medium was added for another 24 hours.

### Cell viability assay

All cell viability assays in this study were performed with a CellTiter-Glo luminescent kit (catalog no. G7571; Promega) according to the manufacturer’s protocols.

### Enzyme-linked immunosorbent assay (ELISA)

PAMs were transfected with siIB169-2 and infected with ASFV-WT at 8 hpt. At 2 hpi, the cells were washed with PBS and incubated with fresh medium. WSL cells were transfected with pFlag-B169L. The expression of IL-1*β* and IL-18 (Ray Biotech, catalog no. ELP-IL1b-1/ ELP-IL 18-A) in the cell culture supernatants were measured by an ELISA kit according to the manufacturer’s instructions at 24 hpi. The concentrations of these cytokines were determined based on standard curves.

### RNA extraction and reverse transcription-quantitative polymerase chain reaction (RT-qPCR)

Total RNA extracted from PAMs was treated with specific inhibitors or infected with ASFV using a Simply P total RNA extraction kit (catalog no. BSC52M1; BioFlux) and then subjected to reverse transcription to generate complementary DNAs (cDNAs) using a FastKing gDNA Dispelling RT SuperMix (catalog no. KR118-02; Tiangen), according to the manufacturer’s protocols. The cDNAs were used to analyze the mRNA expression levels of *B169L, B646L, CP204L, NLRP3, IL-1*β, *IL-18,* and *GAPDH* by RT-qPCR on a QuantStudio system (Applied Biosystems, USA). The mRNA levels of the target genes were normalized to the internal reference gene *GAPDH*, and all the primers used are listed in S1 Table. The relative fold changes in gene expression were determined using the 2^–ΔΔCT^ threshold cycle (CT) method (Livak and Schmittgen, 2001). The ASFV genomic DNA was extracted from the cells and cell supernatants using a MagaBio plus virus DNA purification kit (catalog no. 9109; BioFlux) according to the manufacturer’s protocols. The ASFV genomic DNA copies were quantified by qPCR targeting the *B646L* gene using the primers listed in S1 Table.

### Quantification and statistical analysis

Statistical analysis was performed using GraphPad Prism 8.0 (San Diego, CA). Significant differences between groups were assessed by the Student’s *t* test and one-way ANOVA. All the experiments were performed independently at least three times. The error bars represent the standard deviation (SD) or standard error of the mean (SEM) in each group, as indicated in the figure legends (ns, not significant, *P* ≥ 0.05; *, *P <* 0.05; **, *P <* 0.01; ***, *P <* 0.001). A *P*-value of *<* 0.05 was considered to indicate statistical significance.

## Supporting information

S1 Table Primers used in this study. (DOCX)

S1 Fig Cytotoxicity of the ion-specific inhibitors. HEK293T cells were seeded in 96-well plates and treated with increasing concentrations of ion channel inhibitors, including EIPA, verapamil, amiloride, nifedipine, glibenclamide, 4-AP, BAPTA-AM, EGTA, siB169Ls, thapsigargin (Tg), and 2-APB. After 24 hours, cell viability was tested using the CellTiter-Glo luminescent kit. The significance of the difference between the groups (*n* = 3) was determined using the Student’s *t* test (*, *P *< 0.05; ns, not significant, *P *≥ 0.05).
(TIF)

S2 Fig Amino acid sequence analysis of pB169L. (A) Transmembrane regions of pB169L predicted by DeepTMHMM-1.0 (https://services.healthtech.dtu.dk/services/DeepTMHMM-1.0/), a tool for predicting transmembrane domains in proteins. (B) Multiple sequence alignment of pB169L among the 10 ASFV isolates. The conservation scores based on the biological properties of each amino acid were analyzed by using the Clustal W algorithm.
(TIF)

S3 Fig The hypothetical membrane topology of pB169L. Alphafold2 predicts that the pB169L TM domains form a functional pore as pentamers, but not as tetramers.
(TIF)

S4 Fig Open probability statistics of pB169L and pB169L-TM1. The Open probability of pB169L and pB169L-TM1 in 500 mM: 50 mM KCl solution (-50 mV) were calculated using the Clampfit 10.2 software. The significance of the difference between the groups (*n* = 8) was determined using the Student’s *t* test (*, *P* *<* 0.05, **, *P* *<* 0.01, ***, *P* *<* 0.001; ns, not significant, *P *≥ 0.05.).
(TIF)

S5 Fig Biological characteristics analysis of the ASFV pB169L. (A) Transcriptional dynamics of the ASFV *B169L* gene. The average cycle threshold (Ct) values of *B169L*, *CP204L*, *B646L*, and *GAPDH* in the ASFV HLJ/2018 strain-infected porcine primary alveolar macrophages (PAMs) (MOI = 5) were quantified by reverse transcription-quantitative PCR (RT-qPCR) using the primers targeting *B169L*, *CP204L*, *B646L*, and *GAPDH* at 0, 2, 6, 10, 15, and 24 hours postinfection (hpi). (B) Subviral localization of pB169L. The ASFV HLJ/2018 strain-infected PAMs were fixed with 2% glutaraldehyde at 18 hpi and immunoblotted with rabbit anti-pB169L polyclonal antibodies (PAb) followed by an anti-rabbit IgG antibody conjugated to 5-nm diameter gold particles. The arrowheads indicate the gold particles present on inner envelope of intracellular virus particles. Scale bar, 200 nm. (C and D) Subcellular localization of pB169L. PAMs were infected with ASFV-WT at an MOI of 1. At 24 hpi, the fluorescent signals were analyzed by laser confocal microscopy using rabbit anti-pB169L PAb, mouse anti-CANX/GM130 PAb, and DAPI. Scale bar, 20 µm. The error bars denote the standard errors of the means. The significance of the difference between the groups (*n* = 3) was determined using the Student’s *t* test (*, *P *< 0.05; ns, not significant, *P *≥ 0.05).
(TIF)

S6 Fig ASFV pB169L functions independently of the host IP3R channel. (A) Calcium imaging analysis of pB169L-ΔTM. Representative average traces of Ca^2+^ image of HEK293 cells transfected with pCAGGS, pFlag-B169L, or pFlag-B169L-ΔTM. HEK293 cells were transfected with indicated plasmids. Fura-2 AM was used to monitor ER calcium storage. Ionomycin and thapsigargin (Tg) were added at the time indicated by the arrow. (B) Scatter plot of ionomycin and Thapsigargin induced ratio change of cells transfected with pCAGGS, pFlag-B169L, or pFlag-B169L-ΔTM (*n* > 40). (C) iGLuc luciferase activity analysis of pB169L-ΔTM. HEK293T cells were cotransfected with either pFlag-B169L or pFlag-B169L-ΔTM and NLRP3-dependent iGLuc reporter., and the supernatants were collected to determine luciferase activity at 24 hours posttransfection (hpt). (D) Effects of pB169L-ΔTM on NLRP3 aggregation. HEK293T cells co-transfection of pMyc-NLRP3 and pFlag-B169L-ΔTM, pFlag-B169L or pCAGGS. The fluorescence was observed using a confocal microscope. Scale bar, 20 µm. (E and F) Effects of Tg or 2-APB on the NLRP3-dependent inflammasome activated by pB169L. HEK293T cells were co-transfected with either pFlag-B169L or pB169L-ΔTM-Flag and NLRP3-dependent iGLuc reporter. Tg or 2-APB (an IP3R inhibitor) was added at 12 hpt. The supernatant luciferase activity was examined at 24 hpt. The protein expression was analyzed by Western blotting. (G) Calcium imaging analysis of pB169L-ΔTM upon Tg and 2-APB treatment. HEK293 cells were transfected with indicated plasmids. Fura-2 AM was used to monitor ER calcium storage. Ionomycin or ionomycin and 2-APB was added at the time indicated by the arrow. (H) Calcium imaging analysis of pB169L-ΔTM with Tg and 2-APB treatment. Scatter plot of ionomycin and Tg and 2-APB induced ratio change of cells transfected with pCAGGS, pFlag-B169L, or pFlag-B169L-ΔTM (*n* > 40).
(TIF)

S7 Fig The ASFV pB169L domains and mutations function. (A) Schematic diagram of the pB169L-ΔTM1 or -ΔTM2 domains. The figure was generated by using the online software IBS 2.0 (https://www.ibs.renlab.org/). (B) Expression of the pFlag-B169L mutants. In the presence of the iGLuc-based NLRP3 inflammasome system, HEK293T cells were transfected with different mutant-expressing plasmids. The intracellular expression of the pB169L mutants was evaluated at 24 hours posttransfection (hpi) by Western blotting analysis. (C) Calcium imaging analysis of pB169L mutations. Representative average traces of calcium imaging of HEK293 cells transfected with different point mutated plasmids. HEK293 cells were transfected with different point mutated plasmids. At 24 hpt, the cells were loaded with Fura-2 to monitor ER calcium storage. Ionomycin and Tg were added at the time indicated by the arrow. (D) Scatter plot of ionomycin induced ratio change of HEK293 cells transfected with different point mutated plasmids. Each point represents a single cell.
(TIF)

S8 Fig The B169L protein (pB169L) is essential for IL-1*β* production induced by ASFV infection. (A–F) Effects of ASFV *B169L* gene knockdown on proinflammatory cytokines. PAMs (10^6^ cells/well) were transfected with siRNA-2. At 8 hpt, the cells were infected with ASFV (MOI = 1). The transcription levels of *IL-1β* (A), *IL-18* (B), *NLRP3* (C), and *B169L* (D) were quantified by RT-qPCR at 24 hpi. The supernatants were collected and the secretion of IL-1*β* (E) and IL-18 (F) was detected by enzyme-linked immunosorbent assay (ELISA). (G) Cleavage of GSDMD by pB169L. HEK293T cells were transfected with the designated plasmids, and then analyzed by Western blotting using anti-GSDMD antibodies at 24 hpt.
(TIF)

## References

[1] Ge S, Li J, Fan X, Liu F, Li L, Wang Q, et al. Molecular Characterization of African Swine Fever Virus, China, 2018. Emerg Infect Dis. 2018;24(11):2131–3. doi: 10.3201/eid2411.181274 30141772 PMC6199985

[2] Alejo A, Matamoros T, Guerra M, Andrés G. A Proteomic Atlas of the African Swine Fever Virus Particle. J Virol. 2018;92(23):e01293-18. doi: 10.1128/JVI.01293-18 30185597 PMC6232493

[3] Wang S, Zhang J, Zhang Y, Yang J, Wang L, Qi Y, et al. Cytokine Storm in Domestic Pigs Induced by Infection of Virulent African Swine Fever Virus. Front Vet Sci. 2021;7:601641. doi: 10.3389/fvets.2020.601641 33553280 PMC7862125

[4] Zheng Y, Li S, Li S-H, Yu S, Wang Q, Zhang K, et al. Transcriptome profiling in swine macrophages infected with African swine fever virus at single-cell resolution. Proc Natl Acad Sci U S A. 2022;119(19):e2201288119. doi: 10.1073/pnas.2201288119 35507870 PMC9171760

[5] Liu T, Zhang L, Joo D, Sun SC. NF-κB signaling in inflammation. Signal Transl Target Ther. 2017;2:17023. 10.1038/sigtrans.2017.23 PMC5661633 29158945

[6] Li S, Liang F, Kwan K, Tang Y, Wang X, Tang Y, et al. Identification of ethyl pyruvate as a NLRP3 inflammasome inhibitor that preserves mitochondrial integrity. Mol Med. 2018;24(1):8. doi: 10.1186/s10020-018-0006-9 30134814 PMC6016887

[7] Farag NS, Breitinger U, Breitinger HG, El Azizi MA. Viroporins and inflammasomes: A key to understand virus-induced inflammation. Int J Biochem Cell Biol. 2020;122:105738. doi: 10.1016/j.biocel.2020.105738 32156572 PMC7102644

[8] Nieva JL, Madan V, Carrasco L. Viroporins: structure and biological functions. Nat Rev Microbiol. 2012;10(8):563–74. doi: 10.1038/nrmicro2820 22751485 PMC7097105

[9] Iwatsuki-Horimoto K, Horimoto T, Noda T, Kiso M, Maeda J, Watanabe S, et al. The cytoplasmic tail of the influenza A virus M2 protein plays a role in viral assembly. J Virol. 2006;80(11):5233–40. doi: 10.1128/JVI.00049-06 16699003 PMC1472145

[10] Ruiz A, Guatelli JC, Stephens EB. The Vpu protein: new concepts in virus release and CD4 down-modulation. Curr HIV Res. 2010;8(3):240–52. doi: 10.2174/157016210791111124 20201792 PMC4290667

[11] Steinmann E, Penin F, Kallis S, Patel AH, Bartenschlager R, Pietschmann T. Hepatitis C virus p7 protein is crucial for assembly and release of infectious virions. PLoS Pathog. 2007;3(7):e103. doi: 10.1371/journal.ppat.0030103 17658949 PMC1924870

[12] Wozniak AL, Griffin S, Rowlands D, Harris M, Yi M, Lemon SM, et al. Intracellular proton conductance of the hepatitis C virus p7 protein and its contribution to infectious virus production. PLoS Pathog. 2010;6(9):e1001087. doi: 10.1371/journal.ppat.1001087 20824094 PMC2932723

[13] Breitinger U, Farag NS, Sticht H, Breitinger HG. Viroporins: Structure, function, and their role in the life cycle of SARS-CoV-2. Int J Biochem Cell Biol. 2022;145:106185. doi: 10.1016/j.biocel.2022.106185 35219876 PMC8868010

[14] Xia B, Shen X, He Y, Pan X, Liu F-L, Wang Y, et al. SARS-CoV-2 envelope protein causes acute respiratory distress syndrome (ARDS)-like pathological damages and constitutes an antiviral target. Cell Res. 2021;31(8):847–60. doi: 10.1038/s41422-021-00519-4 34112954 PMC8190750

[15] Moriyama M, Koshiba T, Ichinohe T. Influenza A virus M2 protein triggers mitochondrial DNA-mediated antiviral immune responses. Nat Commun. 2019;10(1):4624. doi: 10.1038/s41467-019-12632-5 31604929 PMC6789137

[16] Ichinohe T, Pang IK, Iwasaki A. Influenza virus activates inflammasomes via its intracellular M2 ion channel. Nat Immunol. 2010;11(5):404–10. doi: 10.1038/ni.1861 20383149 PMC2857582

[17] Ito M, Yanagi Y, Ichinohe T. Encephalomyocarditis Virus Viroporin 2B Activates NLRP3 Inflammasome. PLoS Pathog. 2012;8(8):e1002857. doi: 10.1371/journal.ppat.1002857 22916014 PMC3415442

[18] Gladue DP, Gomez-Lucas L, Largo E, Ramirez-Medina E, Torralba J, Queralt-Martín M, et al. Viroporin-like activity of the hairpin transmembrane domain of African swine fever virus B169L protein. J Virol. 2024;98(8). doi: 10.1128/jvi.00231-24 38980063 PMC11334534

[19] Gong T, Yang Y, Jin T, Jiang W, Zhou R. Orchestration of NLRP3 Inflammasome Activation by Ion Fluxes. Trends Immunol. 2018;39(5):393–406. doi: 10.1016/j.it.2018.01.009 29452983

[20] Cackett G, Matelska D, Sýkora M, Portugal R, Malecki M, Bähler J, et al. The African Swine Fever Virus Transcriptome. J Virol. 2020;94(9):e00119-20. doi: 10.1128/JVI.00119-20 32075923 PMC7163114

[21] Wang Y, Li J, Cao H, Li L-F, Dai J, Cao M, et al. African swine fever virus modulates the endoplasmic reticulum stress-ATF6-calcium axis to facilitate viral replication. Emerg Microbes Infect. 2024;13(1):2399945. doi: 10.1080/22221751.2024.2399945 39230190 PMC11441038

[22] Wang X, Wu J, Wu Y, Chen H, Zhang S, Li J, et al. Inhibition of cGAS-STING-TBK1 signaling pathway by DP96R of ASFV China 2018/1. Biochem Biophys Res Commun. 2018;506(3):437–43. doi: 10.1016/j.bbrc.2018.10.103 30348523

[23] Liu H, Zhu Z, Feng T, Ma Z, Xue Q, Wu P, et al. African Swine Fever Virus E120R Protein Inhibits Interferon Beta Production by Interacting with IRF3 To Block Its Activation. J Virol. 2021;95(18):e0082421. doi: 10.1128/JVI.00824-21 34190598 PMC8387055

[24] Li D, Zhang J, Yang W, Li P, Ru Y, Kang W, et al. African swine fever virus protein MGF-505-7R promotes virulence and pathogenesis by inhibiting JAK1- and JAK2-mediated signaling. J Biol Chem. 2021;297(5):101190. doi: 10.1016/j.jbc.2021.101190 34517008 PMC8526981

[25] Ran Y, Li D, Xiong MG, Liu HN, Feng T, Shi ZW, et al. African swine fever virus I267L acts as an important virulence factor by inhibiting RNA polymerase III-RIG-I-mediated innate immunity. PLoS Pathog. 2022;18(1):e1010270. doi: 10.1371/journal.ppat.1010270 35089988 PMC8827485

[26] Borca MV, O’Donnell V, Holinka LG, Ramírez-Medina E, Clark BA, Vuono EA, et al. The L83L ORF of African swine fever virus strain Georgia encodes for a non-essential gene that interacts with the host protein IL-1β. Virus Res. 2018;249:116–23. doi: 10.1016/j.virusres.2018.03.017 29605728

[27] Silk RN, Bowick GC, Abrams CC, Dixon LK. African swine fever virus A238L inhibitor of NF-kappaB and of calcineurin phosphatase is imported actively into the nucleus and exported by a CRM1-mediated pathway. J Gen Virol. 2007;88(Pt 2):411–9. doi: 10.1099/vir.0.82358-0 17251557

[28] Wang T, Luo R, Zhang J, Lan J, Lu Z, Zhai H, et al. The African swine fever virus MGF300-4L protein is associated with viral pathogenicity by promoting the autophagic degradation of IKKβ and increasing the stability of IκBα. Emerg Microbes Infect. 2024;13(1):2333381. doi: 10.1080/22221751.2024.2333381 38501350 PMC11018083

[29] Huang H, Dang W, Shi Z, Ding M, Xu F, Li T, et al. Identification of African swine fever virus MGF505-2R as a potent inhibitor of innate immunity in vitro. Virol Sin. 2023;38(1):84–95. doi: 10.1016/j.virs.2022.11.009 36442611 PMC10006314

[30] Li J, Song J, Kang L, Huang L, Zhou S, Hu L, et al. pMGF505-7R determines pathogenicity of African swine fever virus infection by inhibiting IL-1β and type I IFN production. PLoS Pathog. 2021;17(7):e1009733. doi: 10.1371/journal.ppat.1009733 34310655 PMC8341718

[31] Xu Y, Wu L, Hong J, Chi X, Zheng M, Wang L, et al. African swine fever virus A137R protein inhibits NF-κB activation via suppression of MyD88 signaling in PK15 and 3D4/21 cells in vitro. Vet Microbiol. 2024;292:110067. doi: 10.1016/j.vetmic.2024.110067 38564905

[32] Zhou P, Dai J, Zhang K, Wang T, Li L-F, Luo Y, et al. The H240R Protein of African Swine Fever Virus Inhibits Interleukin 1β Production by Inhibiting NEMO Expression and NLRP3 Oligomerization. J Virol. 2022;96(22):e0095422. doi: 10.1128/jvi.00954-22 36326277 PMC9683016

[33] Wu P-X, Yang W-P, Feng T, Zhang J, Zhu G-Q, Du X-G, et al. African swine fever virus I177L induces host inflammatory responses by facilitating the TRAF6-TAK1 axis and NLRP3 inflammasome assembly. J Virol. 2025;99(4):e0208024. doi: 10.1128/jvi.02080-24 40135893 PMC11998506

[34] Zhang D, Hao Y, Yang X, Shi X, Zhao D, Chen L, et al. ASFV infection induces macrophage necroptosis and releases proinflammatory cytokine by ZBP1-RIPK3-MLKL necrosome activation. Front Microbiol. 2024;15:1419615. doi: 10.3389/fmicb.2024.1419615 38952452 PMC11215146

[35] Ding J, Wang K, Liu W, She Y, Sun Q, Shi J, et al. Pore-forming activity and structural autoinhibition of the gasdermin family. Nature. 2016;535(7610):111–6. doi: 10.1038/nature18590 27281216

[36] Xia B, Fang S, Chen X, Hu H, Chen P, Wang H, et al. MLKL forms cation channels. Cell Res. 2016;26(5):517–28. doi: 10.1038/cr.2016.26 27033670 PMC4856759

[37] Conos SA, Chen KW, De Nardo D, Hara H, Whitehead L, Núñez G, et al. Active MLKL triggers the NLRP3 inflammasome in a cell-intrinsic manner. Proc Natl Acad Sci USA. 2017;114(6). doi: 10.1073/pnas.1613305114 28096356 PMC5307433

[38] Mao S, Liu X, Wu D, Zhang Z, Sun D, Ou X, et al. Duck hepatitis A virus 1-encoded 2B protein disturbs ion and organelle homeostasis to promote NF-κB/NLRP3-mediated inflammatory response. Int J Biol Macromol. 2024;280(Pt 3):135876. doi: 10.1016/j.ijbiomac.2024.135876 39322136

[39] Triantafilou K, Kar S, van Kuppeveld FJM, Triantafilou M. Rhinovirus-Induced Calcium Flux Triggers NLRP3 and NLRC5 Activation in Bronchial Cells. Am J Respir Cell Mol Biol. 2013;49(6):923–34. doi: 10.1165/rcmb.2013-0032oc 23815151

[40] Triantafilou K, Kar S, Vakakis E, Kotecha S, Triantafilou M. Human respiratory syncytial virus viroporin SH: a viral recognition pathway used by the host to signal inflammasome activation. Thorax. 2013;68(1):66–75. doi: 10.1136/thoraxjnl-2012-202182 23229815

[41] Matamoros T, Alejo A, Rodríguez JM, Hernáez B, Guerra M, Fraile-Ramos A, et al. African Swine Fever Virus Protein pE199L Mediates Virus Entry by Enabling Membrane Fusion and Core Penetration. mBio. 2020;11(4):e00789-20. doi: 10.1128/mBio.00789-20 32788374 PMC7439464

[42] Cuesta-Geijo MÁ, García-Dorival I, Del Puerto A, Urquiza J, Galindo I, Barrado-Gil L, et al. New insights into the role of endosomal proteins for African swine fever virus infection. PLoS Pathog. 2022;18(1):e1009784. doi: 10.1371/journal.ppat.1009784 35081156 PMC8820605

[43] Urquiza J, Cuesta-Geijo MÁ, García-Dorival I, Fernández Ó, Del Puerto A, Díaz JF, et al. Identification of a Potential Entry-Fusion Complex Based on Sequence Homology of African Swine Fever and Vaccinia Virus. Viruses. 2024;16(3):349. doi: 10.3390/v16030349 38543715 PMC10975062

[44] Salas ML, Andrés G. African swine fever virus morphogenesis. Virus Res. 2013;173(1):29–41. doi: 10.1016/j.virusres.2012.09.016 23059353

[45] Zhou P, Li L-F, Zhang K, Wang B, Tang L, Li M, et al. Deletion of the H240R Gene of African Swine Fever Virus Decreases Infectious Progeny Virus Production Due to Aberrant Virion Morphogenesis and Enhances Inflammatory Cytokine Expression in Porcine Macrophages. J Virol. 2022;96(3):e0166721. doi: 10.1128/JVI.01667-21 34787458 PMC8826909

[46] Li S, Ge H, Li Y, Zhang K, Yu S, Cao H, et al. The E301R protein of African swine fever virus functions as a sliding clamp involved in viral genome replication. mBio. 2023;14(5):e0164523. doi: 10.1128/mbio.01645-23 37772878 PMC10653895

[47] Zhao D, Liu R, Zhang X, Li F, Wang J, Zhang J, et al. Replication and virulence in pigs of the first African swine fever virus isolated in China. Emerg Microbes Infect. 2019;8(1):438–47. doi: 10.1080/22221751.2019.1590128 30898043 PMC6455124

[48] Lan J, Luo R, Liu D, Qi C, Song X, Lu Z, et al. A novel high-throughput screen identifies phenazine-1-carboxylic acid as an inhibitor of African swine fever virus replication in primary porcine alveolar macrophages. Vet Res. 2025;56(1):37. doi: 10.1186/s13567-025-01467-2 39923101 PMC11806816

[49] Pan P, Shen M, Yu Z, Ge W, Chen K, Tian M, et al. SARS-CoV-2 N protein promotes NLRP3 inflammasome activation to induce hyperinflammation. Nat Commun. 2021;12(1):4664. doi: 10.1038/s41467-021-25015-6 34341353 PMC8329225

[50] Suzuki T, Orba Y, Okada Y, Sunden Y, Kimura T, Tanaka S, et al. The human polyoma JC virus agnoprotein acts as a viroporin. PLoS Pathog. 2010;6(3):e1000801. doi: 10.1371/journal.ppat.1000801 20300659 PMC2837404

[51] Li Y, Fang L, Zhou Y, Tao R, Wang D, Xiao S. Porcine Reproductive and Respiratory Syndrome Virus Infection Induces both eIF2α Phosphorylation-Dependent and -Independent Host Translation Shutoff. J Virol. 2018;92(16):e00600-18. doi: 10.1128/JVI.00600-18 29899101 PMC6069200

[52] Ao D, Guo H-C, Sun S-Q, Sun D-H, Fung TS, Wei Y-Q, et al. Viroporin Activity of the Foot-and-Mouth Disease Virus Non-Structural 2B Protein. PLoS One. 2015;10(5):e0125828. doi: 10.1371/journal.pone.0125828 25946195 PMC4422707

