# Supplementary material for: The B169L protein of African swine fever virus functions as a viroporin that activates the calcium-mediated inflammasome
Source: PLoS Pathog. 2025 Nov 14;21(11):e1013686. doi: 10.1371/journal.ppat.1013686 (PMC12638030; doi:10.1371/journal.ppat.1013686)
Supplement: S1 Table — (DOCX) [file ppat.1013686.s001.docx]

**S1 Table. Primers used in this study.**

| Primers | Sequence (5′–3′) | Targeting genes |
| --- | --- | --- |
| B169L-1-F | ACAAGAGGCTTTGAATCAGG | ASFV *B169L* |
| B169L-1-R | TCCGTGTCTAATGAGCTAGA |  |
| B169L-2-F | AATAGGGCTACAATGCATAT | ASFV *B169L* |
| B169L-2-R | TCATTACAGCGGTGGTATTA |  |
| B646L(p72)-F | CTGCTCATGGTATCAATCTTATCGA | ASFV *B646L* |
| B646L(p72)-R | GATACCACAAGATC(AG)GCCGT |  |
| CP204L(p30)-F | CGGTAGAATTGTTACGAC | ASFV *CP204L* |
| CP204L(p30)-R | TTCTTGAGCCTGATGTTC |  |
| pGAPDH-F | GAAGGTCGGAGTGAACGGATTT | Porcine *GAPDH* |
| pGAPDH-R | TGGGTGGAATCATACTGGAACA |  |
| IL-1*β*-F | ACCCAAAACCTGGACCTTGG | Porcine *IL-1β* |
| IL-1*β*-R | CATCACAGAAGGCCTGGGAG |  |
| NLRP3-F | CCTTCAGGCTGATTCAGGAG | Porcine *NLRP3* |
| NLRP3-R | GACTCTTGCCGCTATCCATC |  |
| IL-18-F | CGATGAAGACCTGGAATCGG | Porcine *IL-18* |
| IL-18-R | CATCATGTCCAGGAACACTTCTCTG |  |
