# Supplementary figures and images for: The B169L protein of African swine fever virus functions as a viroporin that activates the calcium-mediated inflammasome
Source: PLoS Pathog. 2025 Nov 14;21(11):e1013686. doi: 10.1371/journal.ppat.1013686 (PMC12638030; doi:10.1371/journal.ppat.1013686)

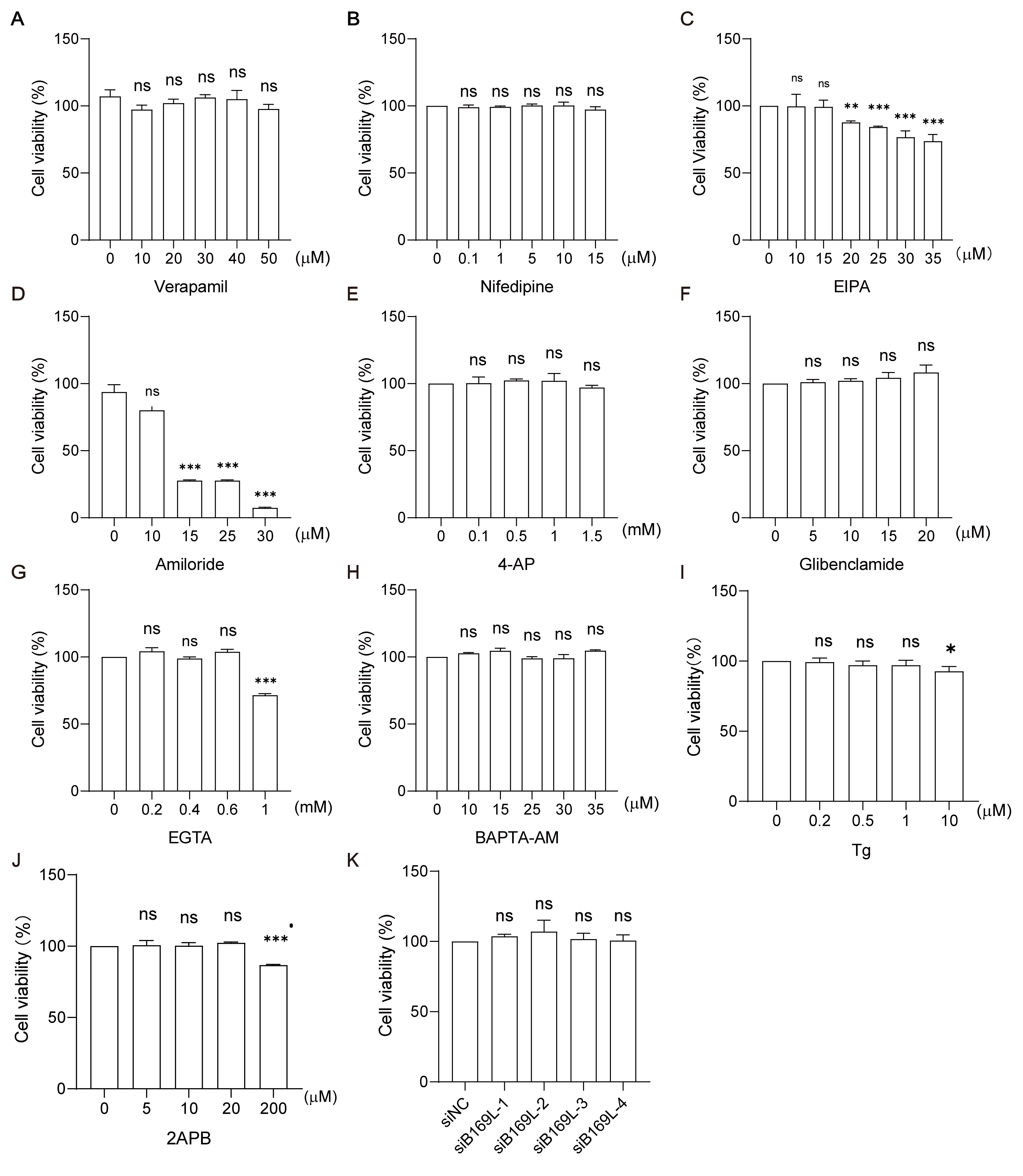

Supplement: S1 Fig — HEK293T cells were seeded in 96-well plates and treated with increasing concentrations of ion channel inhibitors, including EIPA, verapamil, amiloride, nifedipine, glibenclamide, 4-AP, BAPTA-AM, EGTA, siB169Ls, thapsigargin (Tg), and 2-APB. After 24 hours, cell viability was tested using the CellTiter-Glo luminescent kit. The significance of the difference between the groups (n = 3) was determined using the Student’s t test (*, P < 0.05; ns, not significant, P ≥ 0.05). (TIF) [file ppat.1013686.s002.tif]

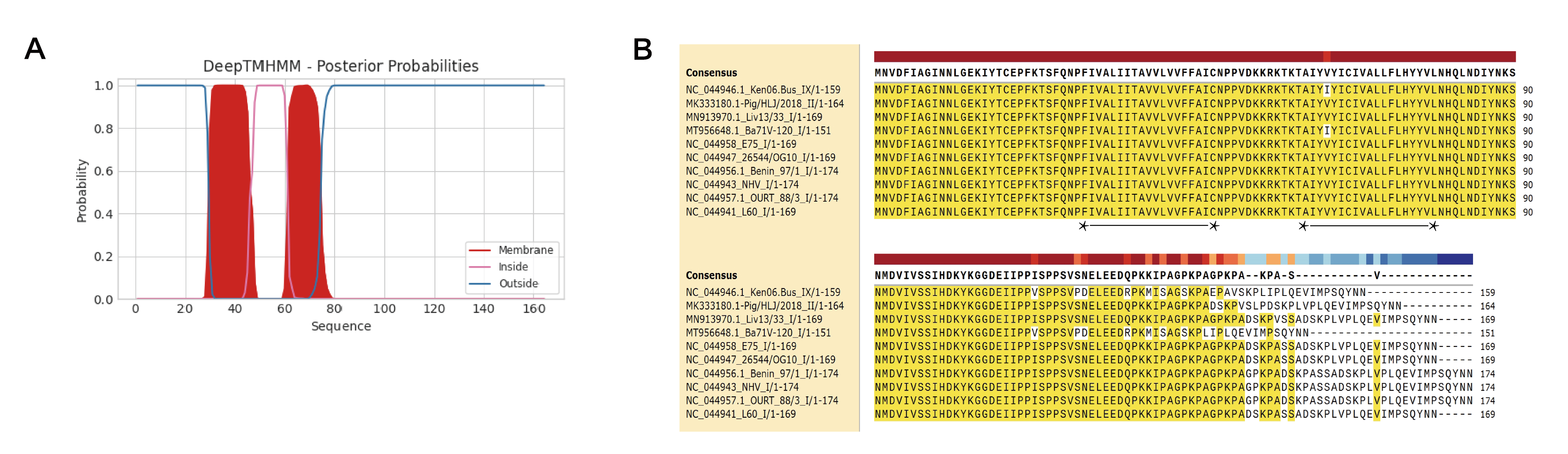

Supplement: S2 Fig — (A) Transmembrane regions of pB169L predicted by DeepTMHMM-1.0 (https://services.healthtech.dtu.dk/services/DeepTMHMM-1.0/), a tool for predicting transmembrane domains in proteins. (B) Multiple sequence alignment of pB169L among the 10 ASFV isolates. The conservation scores based on the biological properties of each amino acid were analyzed by using the Clustal W algorithm. (TIF) [file ppat.1013686.s003.tif]

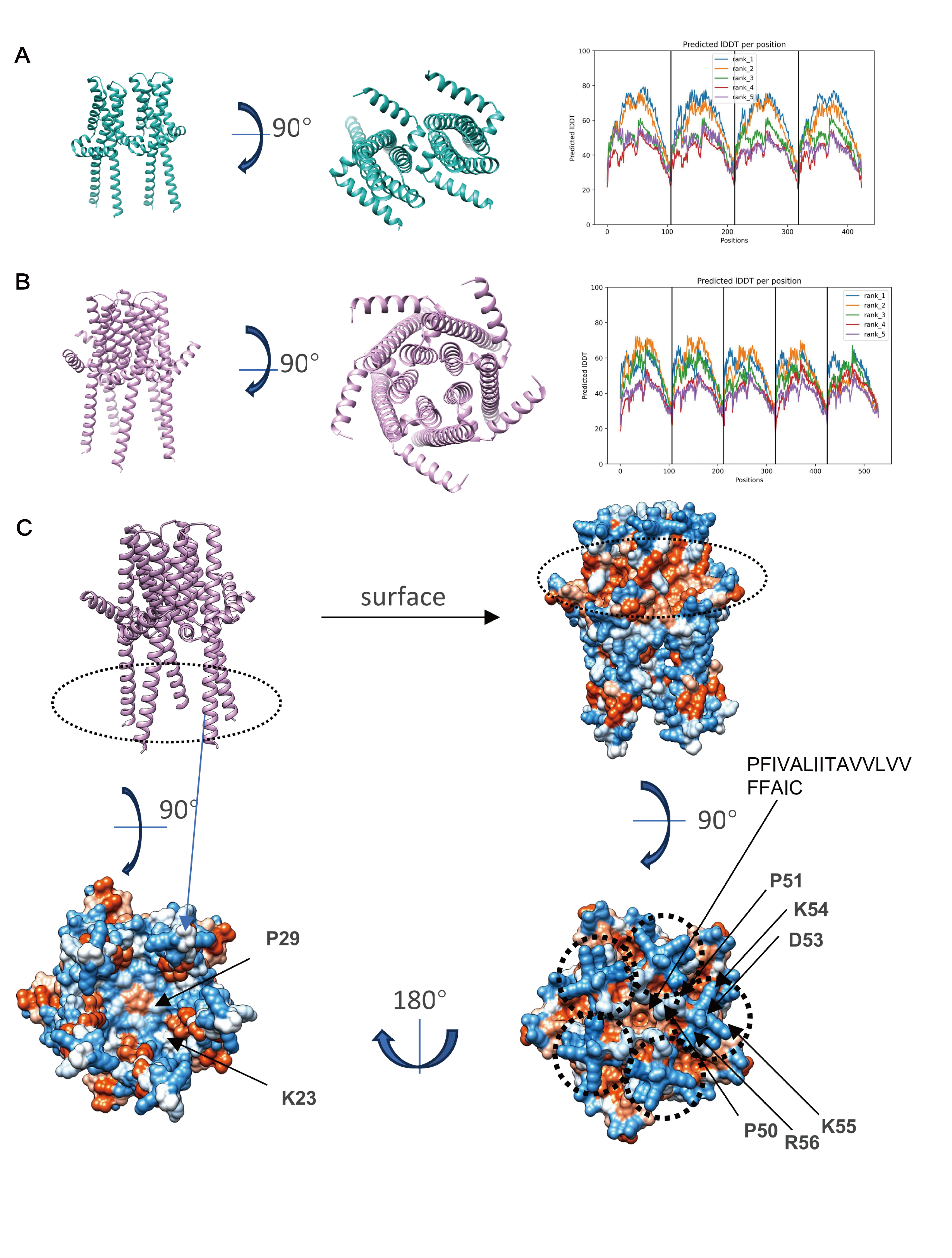

Supplement: S3 Fig — Alphafold2 predicts that the pB169L TM domains form a functional pore as pentamers, but not as tetramers. (TIF) [file ppat.1013686.s004.tif]

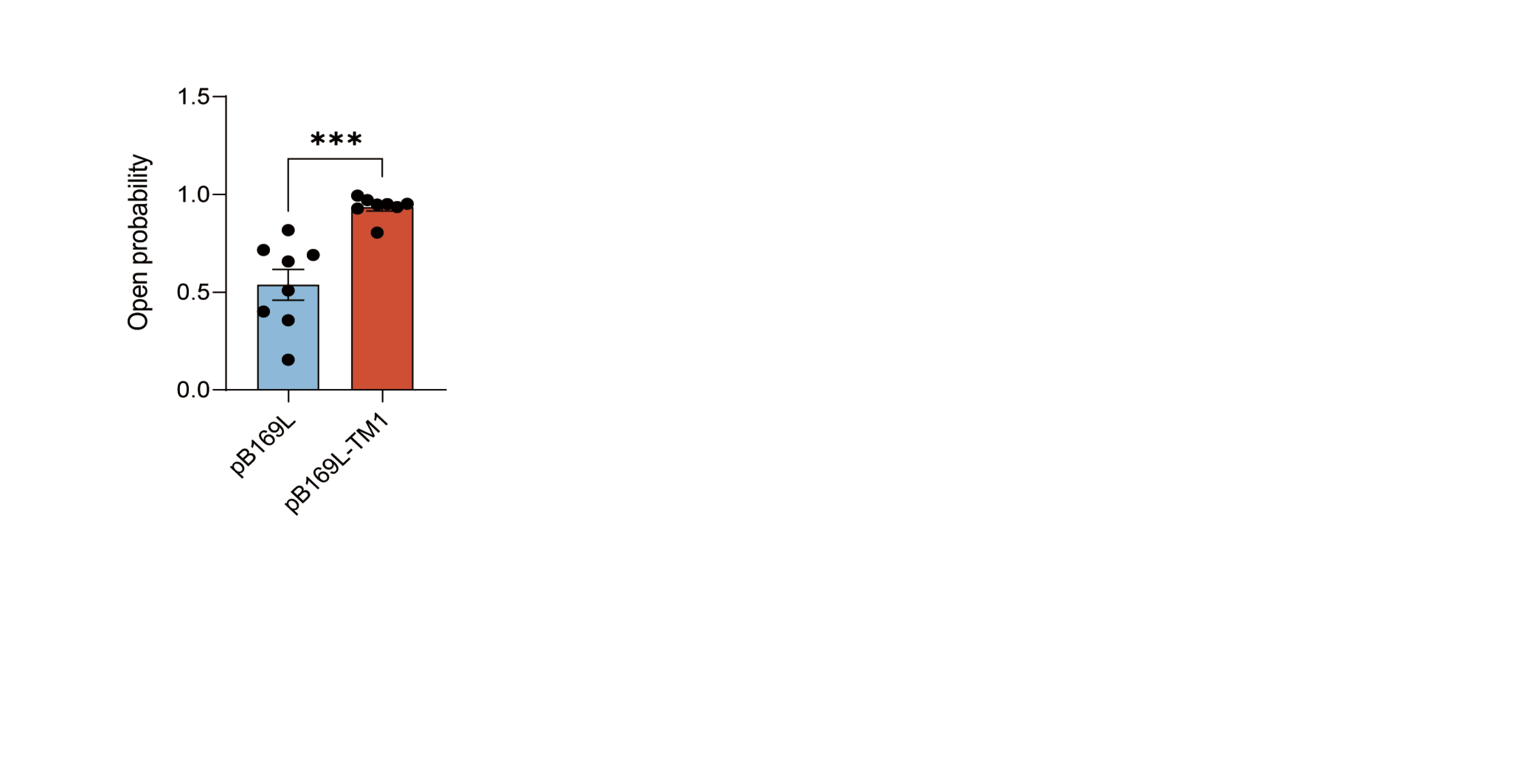

Supplement: S4 Fig — The Open probability of pB169L and pB169L-TM1 in 500 mM: 50 mM KCl solution (-50 mV) were calculated using the Clampfit 10.2 software. The significance of the difference between the groups (n = 8) was determined using the Student’s t test (*, P < 0.05, **, P < 0.01, ***, P < 0.001; ns, not significant, P ≥ 0.05.). (TIF) [file ppat.1013686.s005.tif]

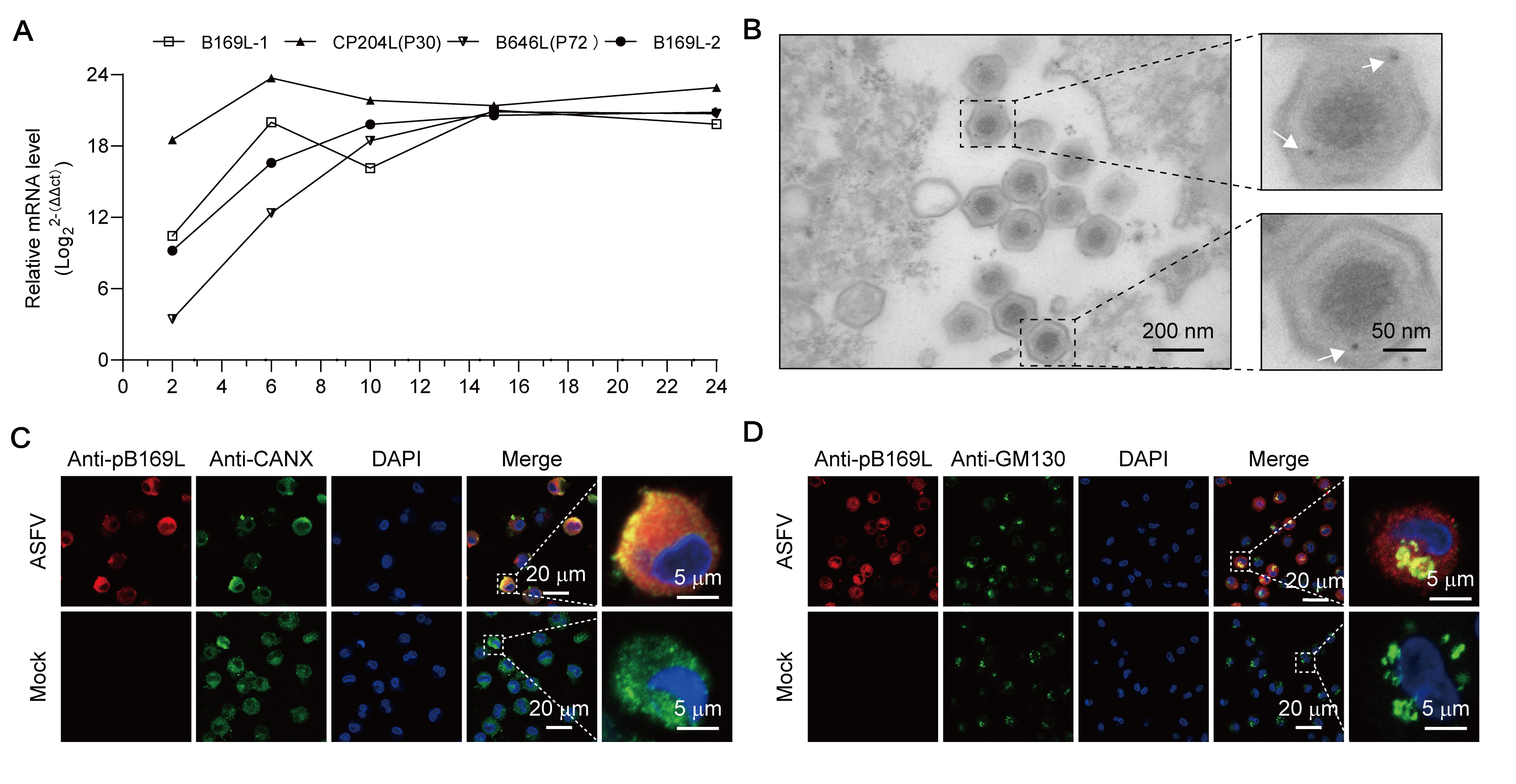

Supplement: S5 Fig — (A) Transcriptional dynamics of the ASFV B169L gene. The average cycle threshold (Ct) values of B169L, CP204L, B646L, and GAPDH in the ASFV HLJ/2018 strain-infected porcine primary alveolar macrophages (PAMs) (MOI = 5) were quantified by reverse transcription-quantitative PCR (RT-qPCR) using the primers targeting B169L, CP204L, B646L, and GAPDH at 0, 2, 6, 10, 15, and 24 hours postinfection (hpi). (B) Subviral localization of pB169L. The ASFV HLJ/2018 strain-infected PAMs were fixed with 2% glutaraldehyde at 18 hpi and immunoblotted with rabbit anti-pB169L polyclonal antibodies (PAb) followed by an anti-rabbit IgG antibody conjugated to 5-nm diameter gold particles. The arrowheads indicate the gold particles present on inner envelope of intracellular virus particles. Scale bar, 200 nm. (C and D) Subcellular localization of pB169L. PAMs were infected with ASFV-WT at an MOI of 1. At 24 hpi, the fluorescent signals were analyzed by laser confocal microscopy using rabbit anti-pB169L PAb, mouse anti-CANX/GM130 PAb, and DAPI. Scale bar, 20 µm. The error bars denote the standard errors of the means. The significance of the difference between the groups (n = 3) was determined using the Student’s t test (*, P < 0.05; ns, not significant, P ≥ 0.05). (TIF) [file ppat.1013686.s006.tif]

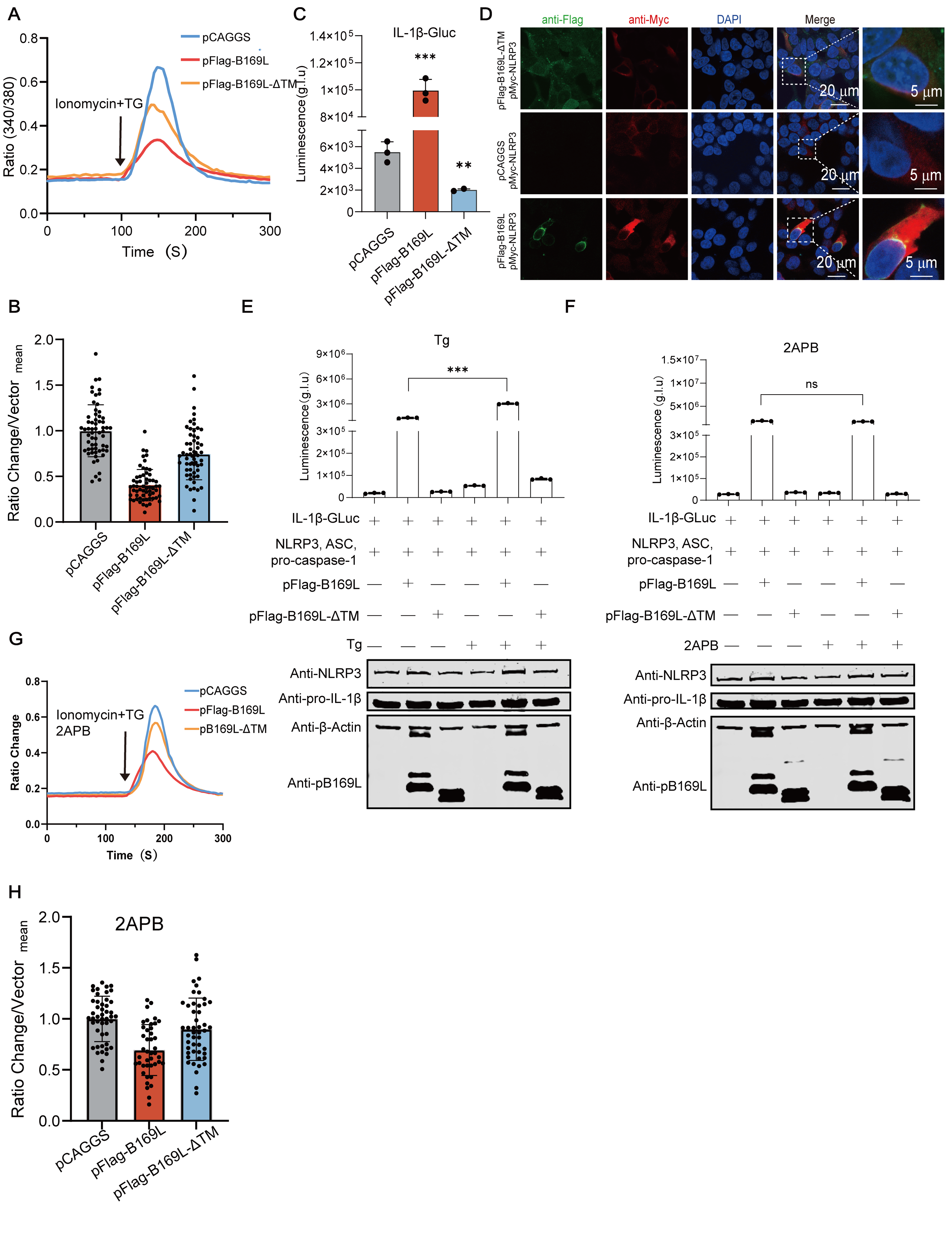

Supplement: S6 Fig — (A) Calcium imaging analysis of pB169L-ΔTM. Representative average traces of Ca2+ image of HEK293 cells transfected with pCAGGS, pFlag-B169L, or pFlag-B169L-ΔTM. HEK293 cells were transfected with indicated plasmids. Fura-2 AM was used to monitor ER calcium storage. Ionomycin and thapsigargin (Tg) were added at the time indicated by the arrow. (B) Scatter plot of ionomycin and Thapsigargin induced ratio change of cells transfected with pCAGGS, pFlag-B169L, or pFlag-B169L-ΔTM (n > 40). (C) iGLuc luciferase activity analysis of pB169L-ΔTM. HEK293T cells were cotransfected with either pFlag-B169L or pFlag-B169L-ΔTM and NLRP3-dependent iGLuc reporter., and the supernatants were collected to determine luciferase activity at 24 hours posttransfection (hpt). (D) Effects of pB169L-ΔTM on NLRP3 aggregation. HEK293T cells co-transfection of pMyc-NLRP3 and pFlag-B169L-ΔTM, pFlag-B169L or pCAGGS. The fluorescence was observed using a confocal microscope. Scale bar, 20 µm. (E and F) Effects of Tg or 2-APB on the NLRP3-dependent inflammasome activated by pB169L. HEK293T cells were co-transfected with either pFlag-B169L or pB169L-ΔTM-Flag and NLRP3-dependent iGLuc reporter. Tg or 2-APB (an IP3R inhibitor) was added at 12 hpt. The supernatant luciferase activity was examined at 24 hpt. The protein expression was analyzed by Western blotting. (G) Calcium imaging analysis of pB169L-ΔTM upon Tg and 2-APB treatment. HEK293 cells were transfected with indicated plasmids. Fura-2 AM was used to monitor ER calcium storage. Ionomycin or ionomycin and 2-APB was added at the time indicated by the arrow. (H) Calcium imaging analysis of pB169L-ΔTM with Tg and 2-APB treatment. Scatter plot of ionomycin and Tg and 2-APB induced ratio change of cells transfected with pCAGGS, pFlag-B169L, or pFlag-B169L-ΔTM (n > 40). (TIF) [file ppat.1013686.s007.tif]

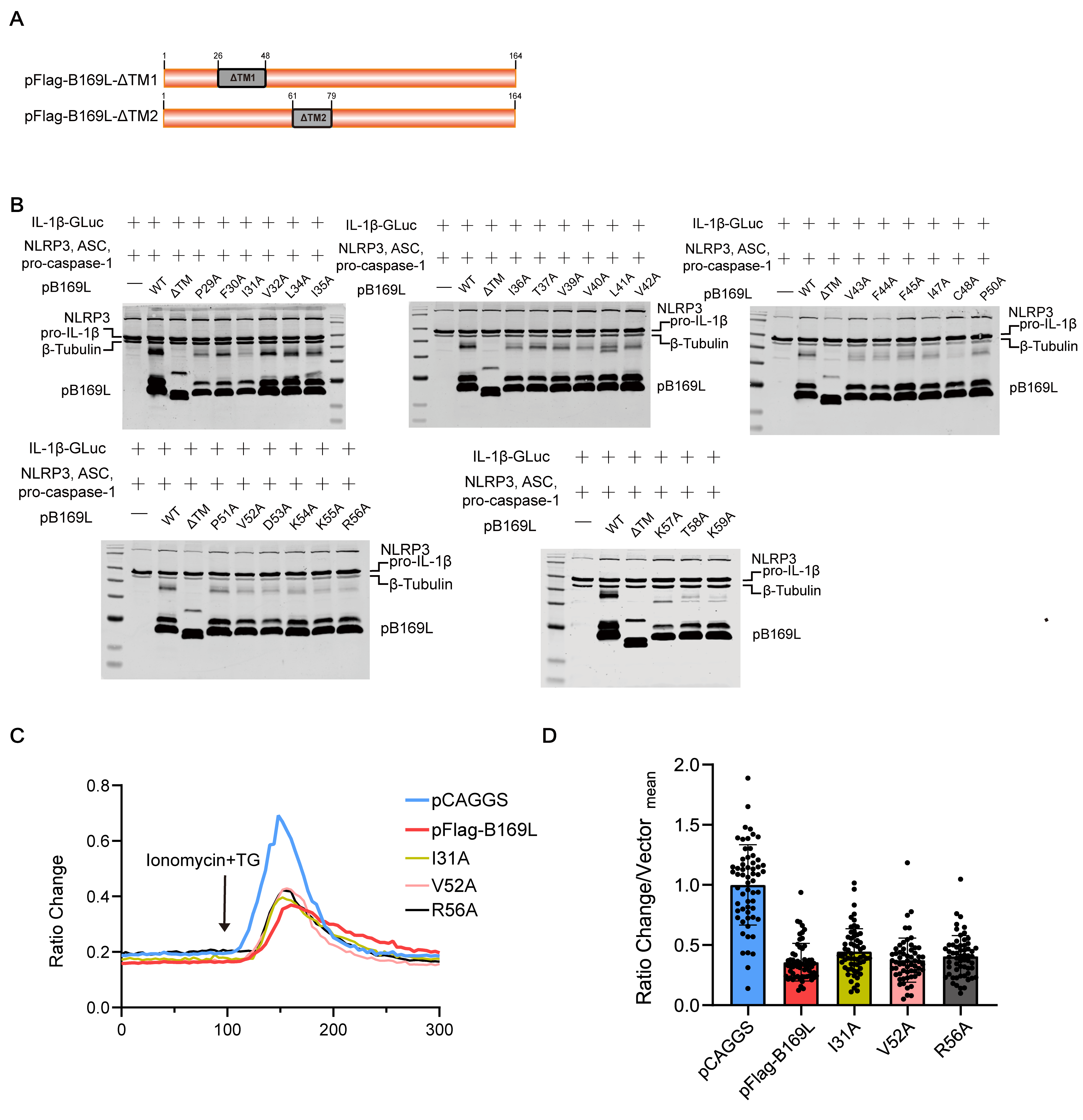

Supplement: S7 Fig — (A) Schematic diagram of the pB169L-ΔTM1 or -ΔTM2 domains. The figure was generated by using the online software IBS 2.0 (https://www.ibs.renlab.org/). (B) Expression of the pFlag-B169L mutants. In the presence of the iGLuc-based NLRP3 inflammasome system, HEK293T cells were transfected with different mutant-expressing plasmids. The intracellular expression of the pB169L mutants was evaluated at 24 hours posttransfection (hpi) by Western blotting analysis. (C) Calcium imaging analysis of pB169L mutations. Representative average traces of calcium imaging of HEK293 cells transfected with different point mutated plasmids. HEK293 cells were transfected with different point mutated plasmids. At 24 hpt, the cells were loaded with Fura-2 to monitor ER calcium storage. Ionomycin and Tg were added at the time indicated by the arrow. (D) Scatter plot of ionomycin induced ratio change of HEK293 cells transfected with different point mutated plasmids. Each point represents a single cell. (TIF) [file ppat.1013686.s008.tif]

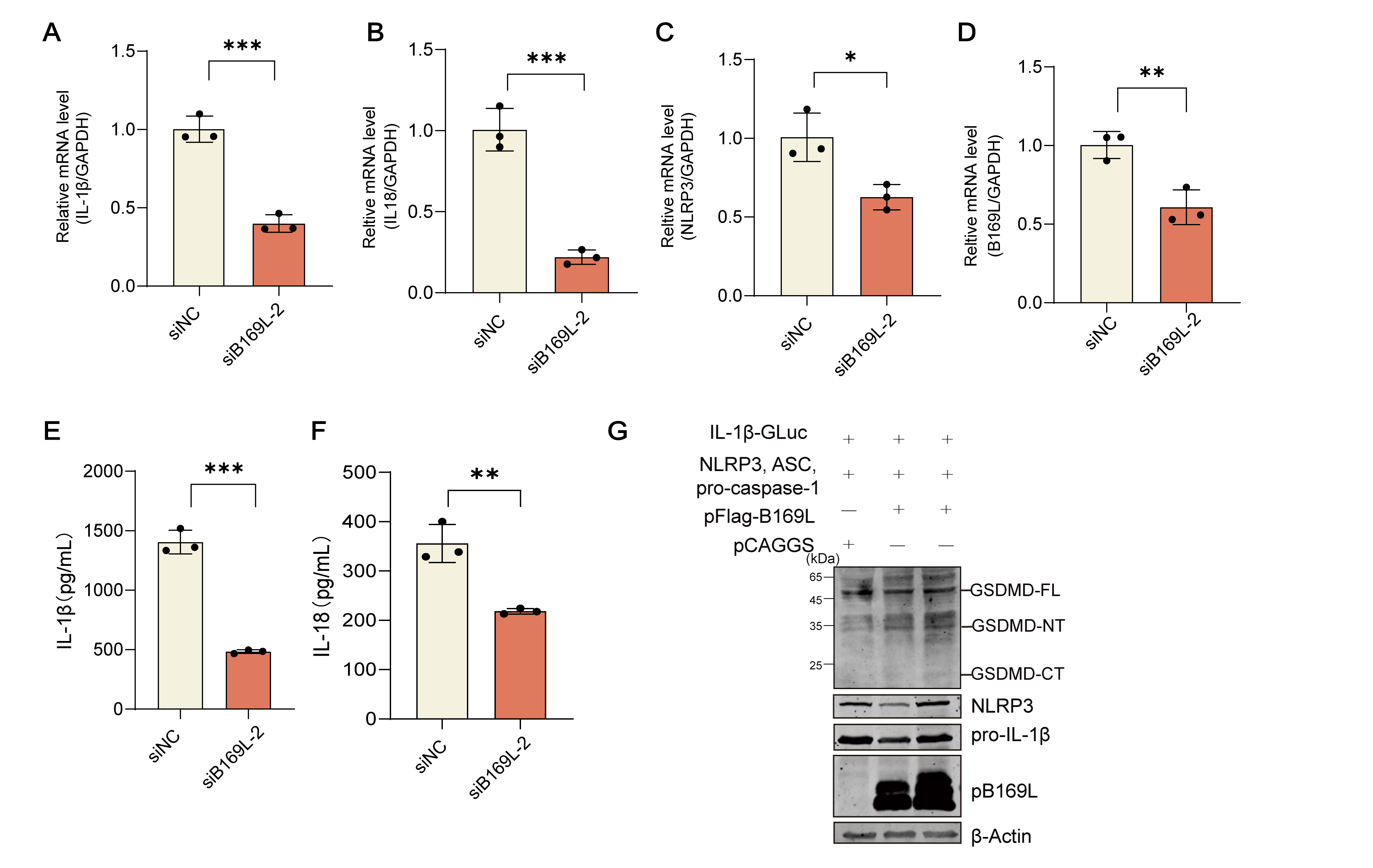

Supplement: S8 Fig — (A–F) Effects of ASFV B169L gene knockdown on proinflammatory cytokines. PAMs (106 cells/well) were transfected with siRNA-2. At 8 hpt, the cells were infected with ASFV (MOI = 1). The transcription levels of IL-1β (A), IL-18 (B), NLRP3 (C), and B169L (D) were quantified by RT-qPCR at 24 hpi. The supernatants were collected and the secretion of IL-1β (E) and IL-18 (F) was detected by enzyme-linked immunosorbent assay (ELISA). (G) Cleavage of GSDMD by pB169L. HEK293T cells were transfected with the designated plasmids, and then analyzed by Western blotting using anti-GSDMD antibodies at 24 hpt. (TIF) [file ppat.1013686.s009.tif]
